# Incidental learning of predictive temporal context within cortical representations of visual shape

**DOI:** 10.1162/imag_a_00278

**Published:** 2024-08-30

**Authors:** Ehsan Kakaei, Jochen Braun

**Affiliations:** European Structural and Investment Funds Graduate School on Analysis, Imaging, and Modelling of Neuronal and Inflammatory Processes, Otto-von-Guericke University, Magdeburg, Germany; Institute of Biology, Otto-von-Guericke University, Magdeburg, Germany; Center for Behavioral Brain Sciences, Otto-von-Guericke University, Magdeburg, Germany

**Keywords:** incidental learning, statistical learning, functional imaging, representational similarity, multi-voxel activity

## Abstract

*Objective:*Incidental learning of spatiotemporal regularities and consistencies—also termed ‘statistical learning’—may be important for discovering the causal principles governing the world. We studied statistical learning of temporal structure simultaneously at two time-scales: the presentation of synthetic visual objects (3 s) and predictive temporal context (30 s) in the order of appearance of such objects.

*Methods:*Visual objects were complex and rotated in three dimensions about varying axes. Observers viewed fifteen (15) objects recurring many times each, intermixed with other objects that appeared only once, while whole-brain BOLD activity was recorded. Over three successive days, observers grew familiar with the recurring objects and reliably distinguished them from others. As reported elsewhere (Kakaei & Braun, 2024), representational similarity analysis (RSA) of multivariate BOLD activity revealed 124 ‘object-selective’ brain parcels with selectivity for recurring objects, located mostly in the ventral occipitotemporal cortex and the parietal cortex.

*Main results:*Here, we extend RSA to the representation of predictive temporal context, specifically “temporal communities” formed by objects that tended to follow each other. After controlling for temporal proximity, we observed 27 ‘community-sensitive’ brain parcels, in which pairwise distances between multivariate responses reflected community structure, either*positively*(smaller distances within than between communities) or*negatively*(larger distances within). Among object-selective parcels, 11 parcels were*positively*community-sensitive in the primary visual cortex (2 parcels), the ventral occipital, lingual, or fusiform cortex (8 parcels), and the inferior temporal cortex (1 parcel). Among non-object-selective parcels, 12 parcels were*negatively*community-sensitive in the superior, middle, and medial frontal cortex (6 parcels), the insula (2 parcels), the putamen (1 parcel), and in the superior temporal or parietal cortex (3 parcels).

*Conclusion:*We conclude that cortical representations of object shape and of predictive temporal context are largely coextensive along the ventral occipitotemporal cortex.

## 1 Introduction

Even when sensory stimuli are experienced passively—without task or reward—they can modify the underlying neural pathways and alter subsequent sensory performance and behavior (e.g.,Conway & Christiansen, 2005;Lengyel et al., 2019;Li & DiCarlo, 2012). This incidental and automatic type of plasticity had been termed ‘statistical learning’ or ‘implicit learning’ (for reviews, seeAslin, 2017;Fiser & Lengyel, 2022;Perruchet, 2019;Perruchet & Pacton, 2006;Saffran & Kirkham, 2018;A. Schapiro & Turk-Browne, 2015). Some theories of cognitive development hypothesize that incidental learning during everyday experience captures the causal processes and relationships underlying sensory observations at a more abstract level (Kemp & Tenenbaum, 2008;Tenenbaum et al., 2011). If so, statistical learning might contribute to higher cognitive function by acquiring the quality of a “structural learning” that could underpin learning from examples, generalizing between domains, or gaining causal insight and understanding (Lake et al., 2017;Shafto et al., 2011).

A well-studied instance of incidental learning is the view-invariance of visual object recognition (for reviews, seeDiCarlo et al., 2012;Gauthier & Tarr, 2016;Logothetis & Sheinberg, 1996). Humans and non-human primates typically recognize visual objects from different viewing directions and distances, presumably relying on characteristic features and/or their spatial relationships. This perceptual invariance can be modified rapidly by the experience of contiguous sequences of different views, demonstrating dependence on learning (Tian & Grill-Spector, 2015;Wallis & Bülthoff, 2001;Wallis et al., 2009). The neural representation of visual shape in the ventral occipitotemporal cortex is similarly view-invariant and equally subject to modification by the recent experience of (natural or unnatural) sequences of views (Jia et al., 2021;Li & DiCarlo, 2008,^2010^,^2012^;Op de Beeck & Baker, 2010;Van Meel & Op de Beeck, 2018,^2020^).

Incidental learning is not limited to individual objects but extends also to spatiotemporal configurations of multiple objects. When human observers experience temporal sequences or spatial arrays of visual objects, task-irrelevant statistical regularities and contingencies are learned rapidly (within minutes), as can be revealed by subsequent behavioral tests (Fiser & Aslin, 2001,^2002^,^2005^;Kakaei et al., 2021;Sáringer et al., 2022;Turk-Browne et al., 2005,^2009^). In non-human primates, the experience of task-irrelevant temporal dependencies modifies object-specific responses of neurons in visual areas of the ventral temporal cortex, but also in multimodal areas of the medial temporal lobe (Erickson & Desimone, 1999;Kaposvari et al., 2018;Meyer et al., 2014;Miyashita, 1988;Sakai & Miyashita, 1991). In human observers, functional imaging evidence reveals that task-irrelevant temporal dependencies can modulate BOLD responses in visually selective areas of the ventral occipital cortex, as well as in multimodal areas such as the medial temporal lobe, hippocampus, and basal ganglia (Gheysen et al., 2011;Giorgio et al., 2018;Hindy et al., 2016;Hsieh et al., 2014;Karlaftis et al., 2019;Turk-Browne et al., 2009,^2010^;A. C. Schapiro et al., 2012;R. Wang et al., 2017). Statistical learning goes beyond first-order dependencies (between immediate temporal neighbors) and extends to higher-order dependencies (between more distant neighbors). For example,A. C. Schapiro et al. (2013,^2016^) demonstrated statistical learning of clusters of dependencies (“temporal communities”) and observed BOLD correlates of this predictive temporal context in associative areas of the frontal and temporal lobes and in the hippocampus, but not in visually selective areas of the ventral occipitotemporal cortex.

Here, we investigate statistical learning by human observers with temporal sequences of visual objects, seeking to compare neural correlates of learning at the levels of individual visual objects and of higher-order temporal dependencies. Unlike previous work, we focus on the visual pathways in the ventral occipitotemporal cortex, the major neural substrate of visual experience and long-term memory (reviewed byBi et al., 2016;Grill-Spector & Weiner, 2014;Kravitz et al., 2013;Weiner & Zilles, 2016). We hypothesize that learning of visual shapes (i.e., spatiotemporal relationships of characteristic features) might interact with the learning of the context in which such shapes appear (i.e., spatiotemporal configurations of distinct shapes) (Miyashita, 1988).

Our visual stimuli were synthetic, three-dimensional objects of unique and characteristic shape that rotated slowly about varying axes. Over three successive sessions/days, observers viewed 15 ‘recurring’ objects approximately 200 times each, as well as 360 ‘non-recurring’ objects once each, while attempting to classify each presented object as either ‘familiar’ (recurring) or ‘novel’ (non-recurring). As reported previously (Kakaei & Braun, 2024;Kakaei et al., 2021), observers quickly gained familiarity with recurring objects and learned to recognize their characteristic shape from all points of view.

We recorded whole-brain BOLD activity during all three sessions/days and analyzed this activity in terms 758 functionally defined brain parcels (Dornas & Braun, 2018), which on average comprised approximately 200 voxels and$1.7cm^{3}$of gray matter. ‘Representational similarity analysis’ (RSA;Haxby, 2012;Kriegeskorte et al., 2008) was used to quantify the information encoded by the BOLD activity of each brain parcel, specifically, the9sof multivariate activity following object presentation. For every brain parcel, this analysis was carried out in a lower-dimensional subspace chosen to maximize the differences between BOLD responses (‘optimal subspace’;Yu & Yang, 2001). A cross-validated analysis identified 124 (of 758) brain parcels that were ‘identity-selective’ in the sense that object identity could be decoded from BOLD activity in the majority of observers: 90 parcels in the ventral occipitotemporal cortex, 28 parcels in the parietal cortex, and 6 parcels in frontal and other regions. The detailed results are reported in a companion study (Kakaei & Braun, 2024).

To investigate the effect of predictive temporal context, we manipulated the order in which objects were presented, adapting the paradigm ofA. C. Schapiro et al. (2013). For three sessions/days, the sequence of recurring objects was generated such as to form ‘temporal communities’ in the sense that every object was likely to be followed by other objects from the same community (“structured condition”). For three further sessions/days, the presentation sequence was fully random so that every object was equally likely to be followed by any other object (“unstructured condition”).

To ascertain the effect of ‘temporal communities’ on multivariate BOLD activity, we analyzed pairwise distances in the ‘optimal subspace’ and established ‘community sensitivity’ in terms of the ratio of average response distances to objects in the same community and in different communities. After controlling for effects of temporal proximity, we established significant ‘community sensitivity’ in 27 of 758 cortical parcels. In particular, we observed*positive*community sensitivity (i.e., smaller distances within than between communities) in 11 ‘identity-selective’ parcels in the ventral occipitotemporal cortex, as well as*negative*community sensitivity (i.e., larger distances within than between communities) in 12 non-identity-selective parcels of the frontal cortex, the insula, the putamen, and the superior temporal or parietal cortex. A further 4 parcels exhibited other combinations of community-sensitivity and identity-selectivity.

We conclude that the ventral occipitotemporal cortex harbors largely coextensive representations of both the identity of objects and of statistical regularities in the order of their appearance.

## 2 Methods

The experimental paradigm and procedure are described in detail elsewhere (Kakaei & Braun, 2024). Here, we only summarize the most pertinent aspects.

### 2.1 Observers and behavior

Eight healthy observers (4 female and 4 male; aged 25 to 32 years) took part in behavioral training (‘sham experiment’, two sessions per observer), the functional imaging experiment (‘main experiment’, six scanning sessions per observer), and a final behavioral assessment (two sessions). All participants were paid and gave informed consent. Ethical approval was granted under Chiffre 30/21 by the ethics committee of the Faculty of Medicine of the Otto-von-Guericke University, Magdeburg.

In both sham and main experiments, observers viewed sequences of 200 recurring and non-recurring objects (see below andFig. 1A) and attempted to classify each object as ‘familiar’ or ‘novel’ (by pressing the appropriate button). Over the course of multiple sessions, observers gradually became familiar with recurring objects and thus became able to distinguish them from non-recurring objects. Objects of the sham experiment were two-dimensional shapes, whereas objects of the main experiment were rotating, three-dimensional shapes (see below andFig. 1A).

**Fig. 1.**
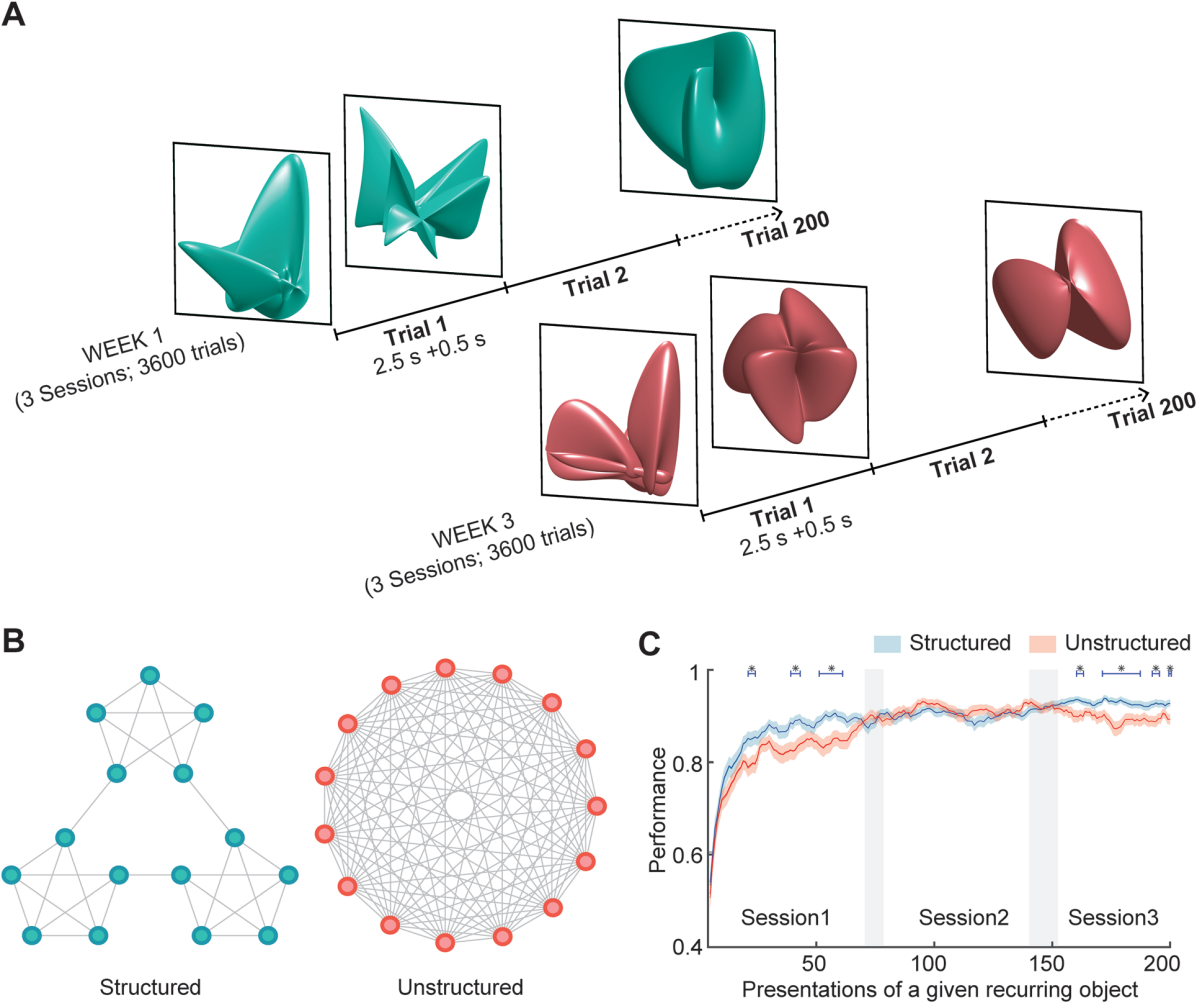
Experimental paradigm. (A) Observers viewed complex, three-dimensional objects that rotated slowly, presented as sequences with 200 trials (2.5 s presentation time and 0.5 s transition time). During the transition, the previous object vanished to the right while the next object approached from the left (please seehttps://learnmem.cshlp.org/content/suppl/2021/04/09/28.5.148.DC1/Supplemental_Movie_S1.mp4). Most objects (180 of 200) recurred multiple times within and between sequences (‘recurring’ objects). The others (20 of 200) were presented only once (‘non-recurring’ objects). Over 3 days/sessions, observers viewed 18 sequences and attempted to classify each object as either ‘familiar’ or ‘unfamiliar’. (B) Sequences were generated from quasi-random walks on either a sparse and modular graph, or a fully-connected and non-modular graph (nodes represent recurring objects, and links represent possible successions). Sequences from the left graph exhibit clustered sequential dependencies (‘structured’). Sequences from the right graph lack such dependencies (‘unstructured’). (C) Over three sessions, observers learned to classify recurring and non-recurring objects as ‘familiar’ and ‘unfamiliar’, respectively. Performance was slightly better with structured than with unstructured sequences. For the present group of observers, the difference was significant during the first and the last sessions (*p< 0.05; FDR corrected). With structured sequences, performance continued to improve slightly from the second to the third session.

The main experiment extended over three successive weeks, with three sessions on separate days of both the first and third week (no sessions took place in the second week). The experiments of the first and third weeks differed in four aspects: sequence type (structured or unstructured), the set of recurring objects, object color (red or blue), and responding hand (left or right). All aspects were counterbalanced across observers. With either responding hand, the index finger responded ‘familiar’ and the middle finger responded ‘unfamiliar’. Observers were not informed about the difference in sequence structure.

After the three scanning sessions of a week, observers participated in an additional behavioral session to confirm that they had, in fact, become familiar with every recurring object. Specifically, they performed a spatial search task in which they pointed out recurring target objects among non-recurring distractor objects (Kakaei et al., 2021). In addition, observers were offered the opportunity to voice anything they might have noticed about the experiment.

### 2.2 Experimental paradigm

Complex three-dimensional objects were computer-generated and presented as described previously (Kakaei et al., 2021). A movie can be viewed under this LINK:https://learnmem.cshlp.org/content/suppl/2021/04/09/28.5.148.DC1/Supplemental_Movie_S1.mp4. All objects were highly characteristic and dissimilar from each other (as confirmed by computational means). Objects were presented every3s, with2.5sviewing and0.5stransition time (Fig. 1A). Objects were shown from all sides and, after appearing at an arbitrary angle, revolved smoothly for one full turn (period2.5s, frequency0.4Hz, angular frequency$144^{\circ }\text{​}/\text{​}s$) about one of several axes in the frontal plane ($-45^{\circ }$,$0^{\circ }$,$45^{\circ }$, clockwise or counter-clockwise). Axes and directions were counterbalanced for each object, and initial viewing angles were chosen randomly (Fig. 1B). All stimuli were generated with MATLAB (The MathWorks, Inc.), presented with the psychophysics toolbox (Brainard, 1997), and viewed in a mirror mounted to the MR head coil (screen resolution960×720pixels, frame rate60Hz, subtending approximately$8^{\circ }\;\times 6^{\circ }$of visual angle, average luminance$50Cd\text{​}/\text{​}m^{2}$, background luminance$5Cd\text{​}/\text{​}m^{2}$). Observers responded with the right or left index finger on an MR-safe response box.

Fifteen objects recurred many times during three sessions (‘recurring’ objects), whereas other objects appeared exactly once (‘non-recurring’ or ‘singular’ objects). As mentioned, observers classified every object as either ‘familiar’ or ‘unfamiliar’ by pressing either the left or right button (counterbalanced) during its presentation. Over the course of three sessions, all observers gradually became familiar with the ‘recurring objects’ (see below). The average time-course of learning, as established by a simplified signal detection analysis, is shown inFigure 1C.

Every session comprised six sequences (‘runs’), each lasting600sand presenting180‘recurring’ and20‘non-recurring’ objects (200objects in total). As there were 15 different recurrent objects, each such object was seen12±1.9times during every sequence. Over the three sessions (or 18 sequences), each recurring object appeared at least190times each (mean±S.D:216±9), whereas non-recurring objects appeared only once. Altogether, there were3,240presentations of recurring objects (3×6×180) and360presentations of non-recurring objects (3×6×180).

### 2.3 Presentation order

To create conditions with and without predictive temporal context (‘structured’ and ‘unstructured’), sequences were generated as quasi-random walks on graphs representing the15recurring objects as nodes and possible continuations as edges (Fig. 1B). Each sequence started at a random node and continued with equal probability on any one of the available edges, except that immediate repetition (X→X) and direct returns (X→Y→X) were not allowed. Although generated randomly, sequences were post-selected to counterbalance the number of appearances of both objects and object pairs (Kakaei et al., 2021). Non-recurring objects were interspersed at random sequence locations.

Structured sequences were generated from the modular graph depicted left inFigure 1B. Note that each object is linked to exactly four other objects (i.e., may be preceded or followed by four other objects). Additionally, links are clustered such as to form three “communities” with five objects each. As a result, the objects from a community tended to follow each other: on average,9±2successive objects derived from the same community, so that these “community episodes” lasted27±6s on average. Moreover, the same objects tended to repeat at short intervals and the expected repetition latency of5.5±15(median and S.D.) was comparatively short.

In structured sequences, the105possible pairings of15objects could be divided into four groups, as illustrated further below inFigure 6A. There were27pairs from the*same*community and*adjacent*on the graph (S*ame*A*djacent*pairs),3pairs from*different*communities and*adjacent*on the graph (DA pairs), as well as3pairs from the*same*community and*non-adjacent*on the graph (SN pairs). Finally,72pairs were from*different*communities and*non-adjacent*on the graph (DN pairs).

Note that onlySApairs andDApairs actually occurred in structured sequences, in the sense that one member occasionally followed the other. Counterbalancing ensured that all objects and all possible object pairs occurred comparably often in presentation sequences (probability approximately1/60). SeeKakaei et al., 2021for further details about the statistics of presentation sequences.

Unstructured sequences were generated from the graph depicted right inFigure 1B. In this graph, each object was linked to all other objects (i.e., it may be preceded or followed by any one of the other objects), so that no sequential dependencies arose. As a result, same objects rarely repeated at short intervals and the expected repetition latency of10.5±11(median and S.D.) was comparatively long.

### 2.4 MRI acquisition

All magnetic-resonance images were acquired on a 3T Siemens Prisma scanner with a 64-channel head coil. Structural images were T1-weighted sequences (**MPRAGE**TR = 2,500 ms, TE = 2.82 ms, TI = 1,100 ms,$7^{\circ }$flip angle, isotropic resolution1×1×1mm, and matrix size of256×256×192). Functional images were T2*-weighted sequences (TR = 1,000 ms, TE = 30 ms,$65^{\circ }$flip angle, resolution of3×3×3.6mm, and matrix size of72×72×36). Field maps were obtained by gradient dual-echo sequences (TR = 720 ms, TE1 = 4.92 ms, TE2 = 7.38 ms, resolution of1.594×1.594×2mm, and matrix size of138×138×72).

### 2.5 fMRI pre-processing

Our approach to fMRI analysis was influenced by recent advances in comparing uni- and multivariate responses of corresponding voxels between different observers (Kumar et al., 2022;Nastase et al., 2019). The*local*correlation structure of voxel response is surprisingly similar in different observers and provides a solid basis for functional parcellation (Dornas & Braun, 2018). Such a parcellation obviates ‘searchlight’ strategies and can define high-dimensional multivariate activity in corresponding ‘parcels’ for different observers.

The fMRI pre-processing procedure was similar to that published previously (Dornas & Braun, 2018). Brain tissues were extracted and segmented using BET (Smith, 2002) and FAST (Zhang et al., 2001). Fieldmap correction, head motion correction, spatial smoothing, high-pass temporal filtering, and registration to structural and standard images were performed with the MELODIC package of FSL (Beckmann & Smith, 2004). Field map correction and registration to structural image were carried out using Boundary-Based Registration (BBR;Greve & Fischl, 2009). MELODIC uses MCFLIRT (Jenkinson et al., 2002) to correct for head motion. Spatial smoothing was performed with SUSAN (Smith & Brady, 1997), with full width at half maximum set at FWHM=5mm. To remove low-frequency artifacts, we applied a high-pass filter of the cut-off frequencyf=0.01Hz, that is, oscillations/events with periods of more than100s were removed. To register the structural image to Montreal MNI152 standard space with isotropic2mmvoxel size, we used FLIRT (FMRIB’s Linear Image Registration Tool;Jenkinson & Smith, 2001;Jenkinson et al., 2002) with 12 degrees of freedom (DOF) and FNIRT (FMRIB’s Nonlinear Image Registration Tool) to apply the non-linear registration. To further reduce artifacts arising from head motion, we applied despiking with a threshold ofλ=100using BrainWavelet toolbox (Patel et al., 2014). Later, we regressed out the mean CSF activity as well as 12 DOF translation and rotation factors predicted by a motion correction algorithm (MCFLIRT). Afterward, the time series of each voxel was whitened and detrended. This resulted in a temporal signal-to-noise ratio (average over time-series, divided by standard deviation over time-series) of approximately 200, with a standard deviation of±30over voxels and of±90over observers.

Finally, the160,099voxels of MNI152 space were grouped into758functional parcels according to the MD758 atlas (Dornas & Braun, 2018). Each functional parcel is associated with an anatomically labeled region of the AAL atlas (Tzourio-Mazoyer et al., 2002) and comprises approximately200voxels or approximately$1.7cm^{3}$of gray matter volume (212±70voxels, range45to462voxels). Parcels were defined for a small population of observers such as to maximize signal covariance within, and minimize covariance between parcels in the resting state. In contrast to other parcellation schemes, this was based exclusively on the (typically strong) functional correlations within each anatomical region and disregarded the (typically weak) correlations between different anatomical regions. The MD758 parcellation offers superior cluster quality, correlational structure, sparseness, as well as consistency with fiber tracking, compared to other parcellations of similar resolution (Albers et al., 2021;Dornas & Braun, 2018).

### 2.6 fMRI data analysis

**Fig. 2.**
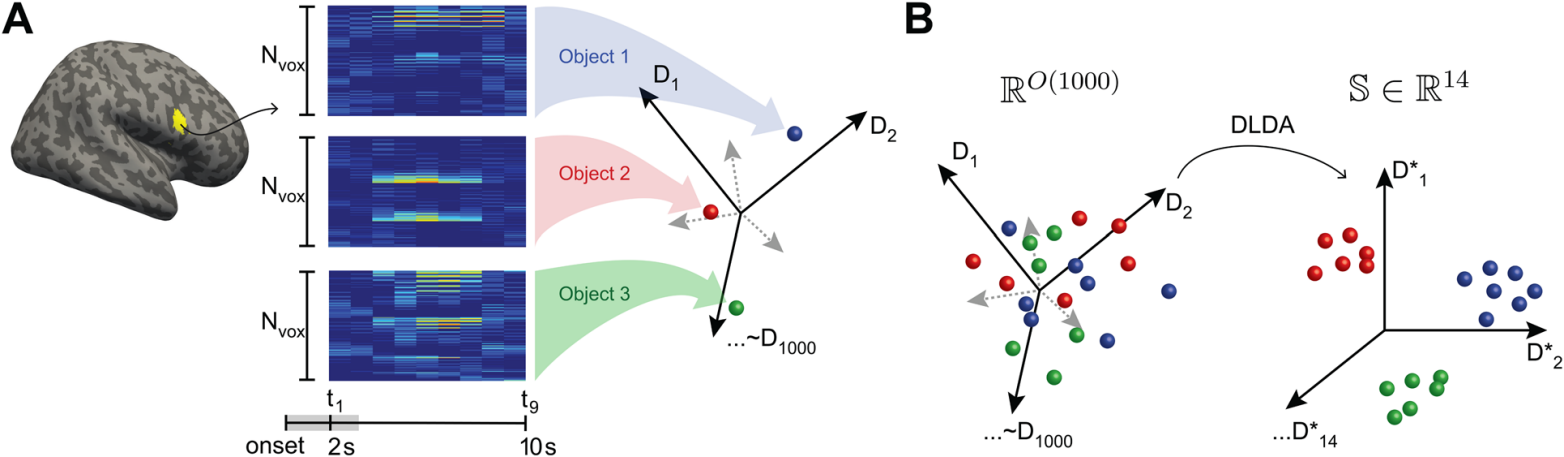
Direct linear discriminant analysis (DLDA) of multivariate BOLD signals. For each observer and functional parcel, we identified a14-dimensional space that optimally discriminated the15classes of activity patterns associated with*recurring*objects. Typically, this space was contained largely within the space of the14principal components (88±5%of variance), but excluded shared variance associated with all object presentations. (A) For a given parcel with$N_{vox}$voxels (yellow: Inf-Front-Oper-R, parcel 146), activity was recorded over9sduring and after object presentation (from2to11safter onset). Each such activity pattern corresponded to a point in a$9\cdot N_{vox}$-dimensional vector space (right), here represented schematically by spheres (red, green, and blue). Images exemplify average responses to three objects with a color scale. (B) In the optimally discriminative subspace,$S\in {\mathbb{R}}^{14}$, Euclidean distance measures the representational similarity of different responses to the same object.

To study the effect of sequence structure on the neural representation of object shape, we extracted the multivoxel activity pattern at$N_{t}=9$time points following object onset. In a functional parcel with$N_{vox}$voxels, this response pattern constituted a point (or vector) in an$N_{dim}$-dimensional space, where$N_{dim}=N_{t}\cdot N_{vox}$(Fig. 2A). Our objective was to compare distances between response patterns to the same objects, to different objects in the same community, and to different objects in different communities, in other words, to analyze representational similarity or dissimilarity in terms of the standardized Euclidean (Mahalanobis) distance between responses in a high-dimensional space (RSA;Kriegeskorte & Diedrichsen, 2019). Over all 758 parcels, response dimensionality was$N_{dim}=1,911\pm 634$(mean and standard deviation), with a range of405to4,158.

To analyze the response variance that discriminates the15recurring objects, we reduced dimensionality with Fisher’s Linear Discriminant Analysis (LDA) for multiple classes to identify the (at most)(κ−1)-dimensional subspaceSthat optimally discriminatesκ=15classes of activity patterns (i.e., responses to the15recurring patterns). Here, optimality is defined as simultaneously minimizing within-class variance and maximizing between-class variance of activity patterns. This approach corresponded to a ‘supervised’ principal component analysis and yielded(κ−1)informative dimensions.

To interpret the results, it is important to appreciate the commonality with principal component analysis (PCA). Over all 758 parcels, the first 14 principal components captured64±5%to the the total response variance following an object presentation. However, about one-third of this variance was shared between presentations and thus uninformative about the identity of the presented object. The 14-dimensional subspacesSidentified by LDA captured the remaining two-thirds (66±5%) of the PCA variance, which were informative about the objects present. In fact, almost all of the subspace variance (88±5%) overlapped with the space of the14leading principal components. Moreover, the subspacesStended to distribute variance more uniformly over dimensions (3±3%per dimension) than principal components did (4±6%per dimension).

This commonality between LDA and PCA explained why subspacesScaptured response variance under all conditions (non-recurring objects, non-selective parcels), not just the conditions for which they had been optimized. A numerically tractable procedure for identifying the optimal subspaceSis available in terms of ‘direct LDA’ or DLDA (Ye et al., 2006;Yu & Yang, 2001). The link github.com/cognitive-biology/DLDA provides a Matlab implementation of DLDA.

The generic nature of subspacesSpermitted us to investigate also the representation of “temporal context” in this way. Specifically, we analyzed the representation of temporal communities with data from structured sequences (8 observers) but performed identical analyses on data from unstructured sequences (8 observers) for comparison. As detailed further below, spurious ‘effects’ of community structure can be observed due to systematic and/or unsystematic fluctuations of responsiveness over time. To guard against such spurious effects, we removed the effects of temporal proximity and verified that our analyses yielded null results with data from unstructured sequences.

#### 2.6.1 Amplitudes, distances, and temporal correlations

Note that the straightforward approach of decoding community identity (i.e., “community selectivity”) would have been confounded by object identity, as any selectivity for “object identity” would necessarily have entailed also some degree of selectivity for “community identity”. To sidestep this issue, we devised a somewhat weaker yet independent measure—“community sensitivity”—which compared pairwise distances between responses to objects within and between communities, as detailed further below.

Activity patterns$x_{jk}$associated with trialskwere analyzed in the maximally discriminative subspaceS. The average normalized amplitude$a_{k}=\sqrt{\frac{1}{\kappa -1}\sum_{j=1}^{\kappa -1}x_{jk}^{2}}$was〈a〉=0.99, and the average normalized distance$d_{kl}=\sqrt{\frac{1}{\kappa -1}\sum_{j=1}^{\kappa -1}{\left(x_{jk}-x_{jl}\right)}^{2}}$between patterns from trialskandlwas〈d〉=1.40. This value corresponds to the normalized distance expected between random patterns, as the average Euclidean distance between two random points, on ann-dimensional hypersphere of unit radius, is

$$d_{ave}=\frac{2^{n}}{\sqrt{\pi }}\frac{\Gamma^{2}\left(\frac{n+1}{2}\right)}{\Gamma \left(n+\frac{1}{2}\right)}$$ (1)

with$d_{ave}\approx 1.4017$forn=14.

On successive trials, activity patterns exhibited a weak temporal correlation, with approximately5%smaller distances at delays below4trials and approximately2%larger distances at delays ranging from6to15trials.Supplementary Figure S2shows the delay-dependent distance between response pairs, as well as the pairwise distance within runs, averaged over all parcels and observers. The delay-dependence of response distances to the same objects was comparable in identity-selective and non-selective parcels, although the delay-dependence of distances to different objects was slightly more pronounced in non-selective parcels. In contrast to multivariate response*distances*, we did not observe any effect of delay on multivariate response*amplitudes*(i.e., we observed neither repetition suppression nor repetition facilitation).

To correct for this temporal correlation, we established for each parcelwthe average delay-dependent distance$T_{w}\left(\Delta \;i\right)={\langle d_{w,u,r}\left(\Delta \;i\right)\rangle }_{u,r}$between patterns with relative delayΔ i, where the average was taken over subjectsuand runsr. The time-course$T_{w}$allowed us to subtract the average effect of temporal correlation by computing residual distance$d_{w,u,r}^{corrected}\left(\Delta \;i\right)=d_{w,u,r}\left(\Delta \;i\right)-T_{w}\left(\Delta \;i\right)+{\langle T_{w}\left(\Delta \;i\right)\rangle }_{\Delta \;i}$, where${\langle T_{w}\left(\Delta \;i\right)\rangle }_{\Delta \;i}$is the average value over delaysΔi.

#### 2.6.2 Measure of identity-selectivity

Selectivity for object identity was quantified in terms of “classification accuracy”,$\alpha^{identity}$, which was defined as the probability that a multivariate response was classified correctly on the basis of distance to class centroids. To test for statistical significance, we relied on the “minimum accuracy” over all observers or data sets (Allefeld et al., 2016). Further details are provided in the companion paper (Kakaei & Braun, 2024).

#### 2.6.3 Geometry of temporal community representations

To assess the representation of community structure, we compared pairwise distances between responses to objects within and between communities for each parcelw. Specifically, we first obtained pairwise distances$d_{ij}$and sorted them into two groups: within-community distances with average$D_{w}^{W}=\langle d_{ij}(ij|i,j\in L)\rangle$and between-community distances with average$D_{w}^{B}=\langle d_{ij}(ij|i\in L,j\in K,L\neq K)\rangle$. Then, we established the signed difference$\Delta_{w}^{BW}=\langle D_{w}^{B}\rangle -\langle D_{w}^{W}\rangle$, which we termed “community sensitivity”, and assessed the statistical significance of$\Delta_{w}^{BW}$with a two-sample t-test. After correcting for false discovery (Benjamini & Hochberg, 1995), we summarized the results for each parcel in terms of*t*-statistics$t^{BW}$.

A similar procedure was used to assess differences between classes of object pairs. Specifically, for every parcel, we established the average pairwise distance$D_{w}$(averaged over all pairs and all observers) for different classes of object pairs: same community & adjacent (SA), same community & non-adjacent (SN), different communities & adjacent (DA), and different communities & non-adjacent (DN). The resulting values were termed$D_{w}^{SA}$,$D_{w}^{DA}$,$D_{w}^{SN}$, and$D_{w}^{DN}$. The statistical significance was assessed by comparing the observed values to the pairwise distance$D_{w}^{diff}$, which contains pairwise distances of all 4 types of object pairs, by a two-sampledt-test. The results were summarized in terms of t-statistics$t_{w}^{SA}$,$t_{w}^{DA}$,$t_{w}^{SN}$, and$t_{w}^{DN}$. The behavioral evidence (Kakaei et al., 2021) informed our a-prior hypothesis that SA pairs might be more similar, and NA pairs more dissimilar, than the overall average. A further a-priori hypothesis was that DA pairs might be more dissimilar, as they involve the “linking objects” that mark transitions between different communities.

Note that response distances within and between communities are confounded by temporal proximity because responses*within*communities tend to have shorter relative latencies than responses*between*communities (A. C. Schapiro et al., 2013). To assess the degree to which temporal proximity contaminates the observed community signal, we repeated the analysis of community representations for different ranges of temporal latencies. Specifically, we recalculated the average pairwise distances$D_{w}^{between}$and$D_{w}^{within}$, and the corresponding$t_{w}^{BW}$for object pairsi,jwhose relative latencies$\tau_{ij}$where bounded from below by$\tau_{LB}\leq \tau_{ij}$and from above by sequence termination, with the lower bound ranging over$\tau_{LB}\in \left\{1,\ldots ,30\right\}$. Thet-statistics of response pairs with bounded latencies and their corresponding p-values, corrected for false discovery rate, will be denoted as$t^{BW}\left(\tau_{LB}\right)$and$P^{BW}\left(\tau_{LB}\right)$, respectively.

To assess whether community representations are consistent over different latency ranges, we examined how$t^{BW}\left(\tau_{LB}\right)$changes with its lower bound$\tau_{LB}$. Specifically, for each parcel, we defined a consistency measure$\tau_{sig}$as the highest lower bound at which$t^{BW}\left(\tau_{sig}\right)$remains significant. We considered a parcel as*‘community sensitive’*only if$\tau_{sig}\geq 30$. In other words, a ‘community sensitive’ parcel exhibited significant between-community separability$t^{BW}$for all lower bounds$\tau_{LB}\in \left\{1,\ldots ,30\right\}$. This ruled out the possibility that community sensitivity was a spurious effect of temporal proximity (which was strongest at shorter latencies).

#### 2.6.4 Statistical power

The representational similarity analysis concerning object identity described inKakaei and Braun (2024)was based on approximately216object responses (18sequences with approximately12recurrences of each object) from each of16data sets, affording approximately370,000representational distances for each of the105object pairs. In contrast, the assessment of representational similarity concerning community was based on approximately120community episodes (18sequences with approximately6recurrences of each community) from each of8data sets, affording approximately57,000representational distances for each of the3community pairs. Hence, the number of independent pairwise observations about identity was approximately225times larger than the number about community. Accordingly, on purely statistical grounds, the sensitivity of our paradigm for detecting community sensitivity is expected to be approximately15times*lower*than for detecting identity selectivity.

As a consequence of this statistical disparity, we were unable to establish the temporal development of “community sensitivity” over the 3 days/sessions (seeSupplementary Fig. S8). For “object identity”, we could demonstrate temporal developments not only over days/sessions but even over individual runs (Kakaei & Braun, 2024).

#### 2.6.5 Dimensional reduction

To visualize the representational geometry of community structure in two dimensions, we calculated a distance matrix$D_{w,u,r}\left(i,j\right)=\langle d_{ij}\rangle$of response distances corrected for temporal proximity within each runr, for every parcelwand observeru. Averaging over the runs produced matrices$D_{w,u}$of size15×15of the average distances between the 15 recurring objects in the discriminative subspaceS.

As we did not expect different observers to exhibit comparable activity patterns and distance matrices, we did not wish to average these matrices directly. To sidestep the difficulty, we permuted the object order of the matrix$10^{4}$times while maintaining graph structure (adjacency and module membership), to first obtain an ensemble average matrix${\bar{D}}_{w,u}$for each observer, and finally the observer average${\bar{\bar{D}}}_{w}$of ensemble averages.

Using multidimensional scaling (Matlab function*mdscale*), we converted the observer average matrix${\bar{\bar{D}}}_{w}$to a two-dimensional map of15locations approximating these pairwise distances. These maps reveal the average response distance between objects within and between communities, as well as the average distance between ‘linking’ and other objects. Note that the three-fold rotational symmetry of these maps is owed to the permutation procedure.

## 3 Results

### 3.1 Behavior

Observers readily became familiar with recurring objects, as confirmed by the time course of performance in classifying objects as ‘familiar’ (recurring) or ‘novel’ (non-recurring), which exceeded 75% correct after one session and approached 90% performance after two further sessions (Fig. 1C). Typically, the classification of a particular object changes from ‘novel’ to ‘familiar’ at a particular point in time (“onset of familiarity”). After the experiment, several observers mentioned having invented linguistic labels for each recurring object (‘anchor’, ‘butterfly’, ‘hedgehog’, etc.). Some observers mentioned noticing that objects repeated in close temporal proximity in the ‘structured condition’. However, no observer mentioned noticing that the recurring objects formed three distinct “communities”.

In the structured condition, familiarity increased slightly faster and “onsets of familiarity” occurred somewhat sooner. Specifically, performance was slightly but significantly higher during much of the first and third sessions, and comparable in the second session (Fig. 1C). Moreover, after an object became familiar, the next object to do so was significantly more likely than chance to be a ‘same adjacent’ (SA) object and significantly less likely to be a ‘different non-adjacent’ (DN) object. Specifically, the frequency of successive onsets of familiarity was elevated by0.15(p<0.05) for SA pairs, and reduced by−0.15(p<0.05) for DN pairs, but did not differ significantly for either DA pairs (“linking objects”) or SN pairs.

Average reaction times mirrored the performance results in that they were higher before than after the “onset of familiarity” (p<0.01). In the structured condition, reaction times for linking objects (members of DA pairs) and internal objects (all others) did not differ significantly during either the first, second, or third session (p<0.01). Thus, the behavioral effects of sequence structure did not extend to reaction times. This was consistent with the behavioral results reported previously (Kakaei et al., 2021).

### 3.2 Representation of temporal community structure

To assess the effects of temporal community structure, we analyzed the multivariate BOLD activity of each brain parcel over9s(or9TR), starting with the onset of object presentation. Specifically, we analyzed linear distances between multivariate responses after reducing the dimensionality of the originallyO(1,000)-dimensional responses to the14dimensions of an ‘optimal subspace’S. We chose this subspace such as to maximize the discriminability of responses to different recurring objects, using Fisher’s linear discriminant analysis (LDA). Unlike principal component spaces, the optimal subspaces disregarded variance that was shared between responses to different objects and emphasized variance that distinguished responses to different objects. The dimensionality of the subspace (14) reflected the number of recurring objects (15) and was large enough to capture the major part of the response variance.

Over all 758 parcels, the first 14 principal components captured64±5%of the total response variance following an object presentation. However, about one-third of this variance was shared between presentations of different objects and was thus uninformative about the objects. The 14-dimensional subspacesSidentified by LDA captured the remaining two-thirds (66±5%) of the PCA variance, which were informative about the objects present. Moreover, the subspacesStended to distribute variance more uniformly over dimensions (3±3%per dimension) than principal components did (4±6%per dimension).

**Fig. 3.**
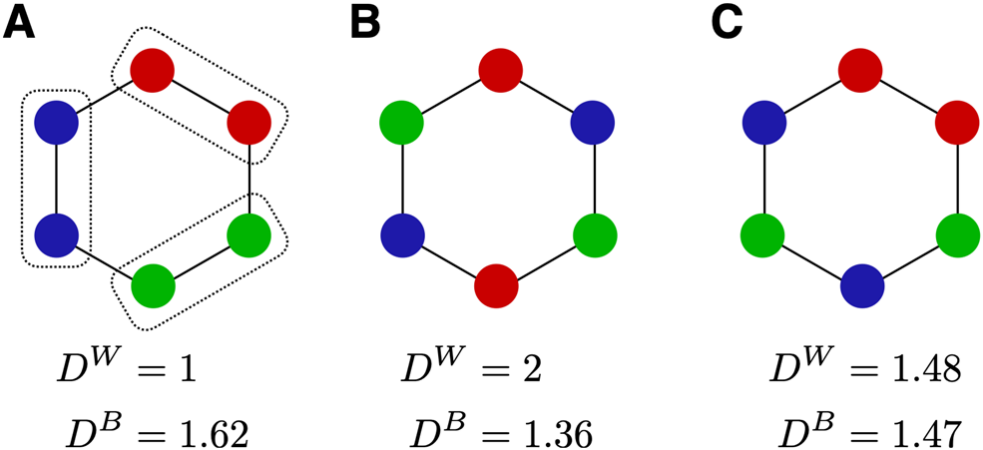
Possible representations of temporal community structure (highly schematic). Disks represent multivariate responses to 6 objects, and two-dimensional distances represent multivariate distance. Colors represent temporal communities. In each case, the average distance within and between communities is provided ($D^{W}$and$D^{B}$, respectively). (A) Responses to objects are closer within than between communities (dotted boxes). (B) Responses to objects are further apart within than between communities. (C) Responses to objects are, on average, comparably distant within and between communities.

In principle, response distances could have reflected temporal community structure in different ways, as illustrated schematically inFigure 3. For example, responses to objects in the same community could be systematically*closer together*than to objects in different communities, indicating greater representational similarity (‘positive sensitivity’;Fig. 3A). Alternatively, responses to objects in the same community could be systematically*further apart*than to objects in different communities, indicating less representational similarity (‘negative sensitivity’;Fig. 3B). A third possibility would be no systematic relationship between response distance and community membership (Fig. 3C). Optimal subspaces were chosen such as to maximize distances between objects regardless of temporal community and thus favored neither possibility over another. In fact, optimal subspaces were computed in the same way whether or not temporal communities were present (structured and unstructured conditions).

A difficulty in assessing community sensitivity is that it is confounded by the known temporal auto-correlation of multivariate activity (A. C. Schapiro et al., 2013). As illustrated inFigure 4A, pairwise distances were computed for all observers, runs, and parcelsw, to obtain average pairwise distance$D_{w}^{within}$communities, average pairwise distance$D_{w}^{between}$between communities, and the average separability$\Delta_{w}^{BW}=D_{w}^{between}-D_{w}^{within}$. For every parcelw, we established “community sensitivity” by assessing whether or not$\Delta_{w}^{BW}$values differed significantly from zero with t-statistic$t^{BW}$. Two measures were taken to correct for temporal auto-correlations and to dissociate community sensitivity and temporal proximity (seeSection 2.6.1). Firstly, for each raw pairwise distance and its latency, we computed a residual pairwise distance by subtracting the average distance at that latency. Secondly, we compared the t-statistic$t^{BW}$for subsets of pairwise distances covering different temporal latency ranges ($\tau_{LB}\leq \tau \leq 30;\tau_{LB}\in \left\{1,\ldots ,30\right\}$). For all parcels, significance decreased monotonically when lower bound$\tau_{LB}$was raised and shorter latencies were progressively excluded. Thus, the situation was summarized by the*largest*value of$\tau_{LB}$at which$t^{BW}$statistic was significant, which value was termed$\tau_{sig}$. A high value of$\tau_{sig}$indicated significance over all latency ranges, both including shorter latencies (low values of$\tau_{LB}$) and excluding shorter latencies (high values of$\tau_{LB}$). A low value of$\tau_{sig}$indicated significance only for ranges that included shorter latencies (low values of$\tau_{LB}$).

**Fig. 4.**
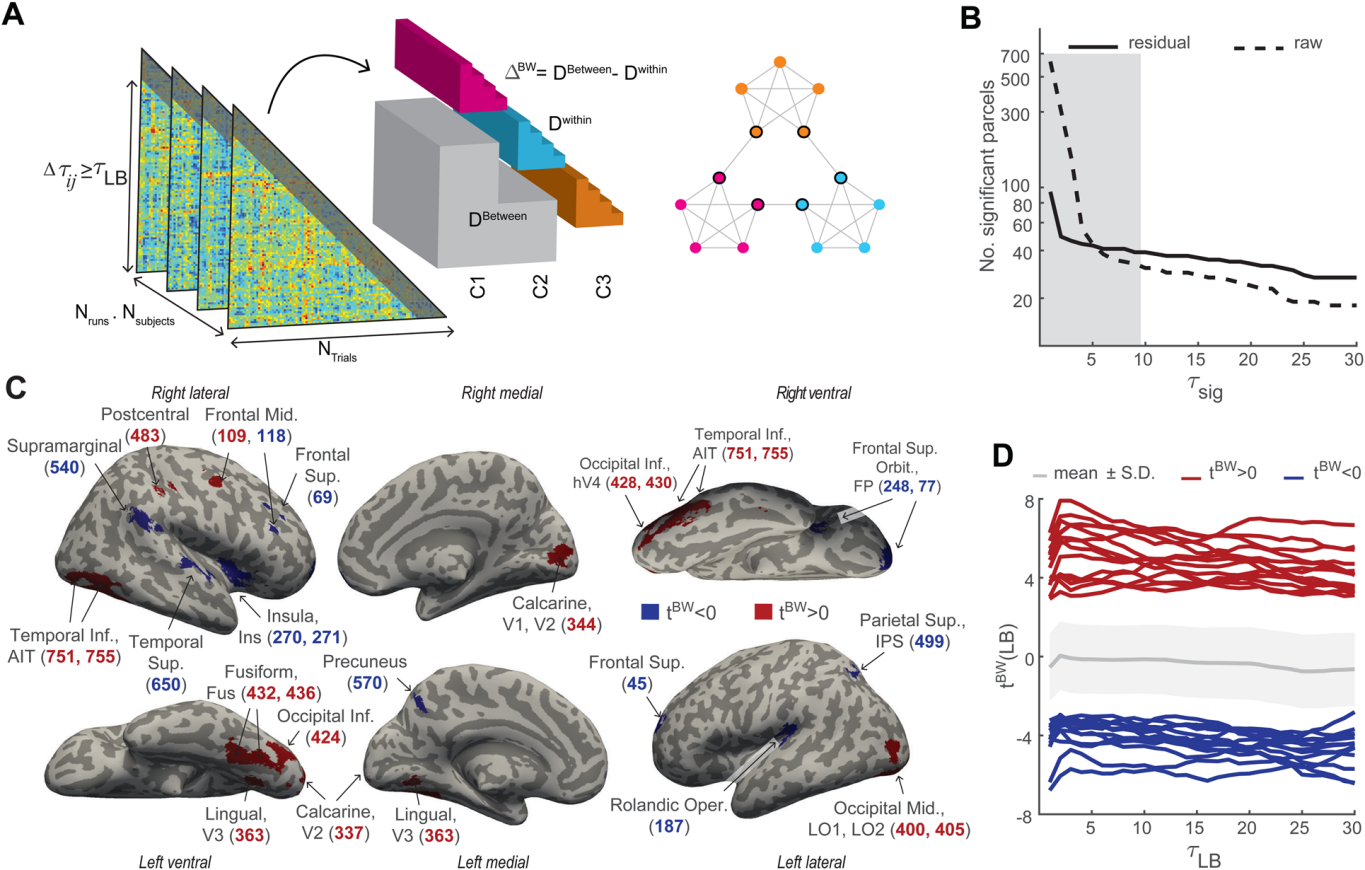
Distribution of sensitivity to temporal community structure. (A) Pairwise distances between object responses (triangular matrices) were corrected for the average auto-correlation, thresholded by latency$\tau_{ij}$(lower bound$\tau_{LB}$, indicated by shading), and sorted into different subsets—within-community pairs (cyan, magenta, orange) and between-community pairs (grey)—according to object positions on the modular path (right). For the average signed difference$\Delta^{BW}$, statistic$t^{BW}$was computed. (B) Number of parcels with consistent significance up to$\tau_{sig}$for residual (solid) and raw (dashed) pairwise distances. The average duration of a community visit was9.4±0.15(gray shading). (C) Representation of ‘temporal communities’ by parcels with consistently significant$\Delta^{BW}$. In 14 parcels (red), between-community pairs are significantly more separable ($t^{BW}>0$, correctedp<0.05) over all latency ranges whereas, in 13 parcels (blue), within-community pairs are more separable ($t^{BW}<0$) over all ranges. Labeling indicates parcels in visual cortex (V1, V2, V3, hV4), lateral occipital cortex (LO1, LO2), fusiform and lingual gyrus (Fus, Lin), anterior inferiotemporal cortex (AIT), intraparietal sulcus (IPS), superior temporal cortex, supramarginal gyrus, medial frontal cortex, precuneus, insula (Ins), Rolandic operculum, precentral cortex, and frontal pole (FP). (D) Between-community separability$t^{BW}$for different latency ranges (lower bound$t^{BW}$), for ‘community-sensitive’ parcels with positive$t^{BW}>0$(red) and negative$t^{BW}<0$(blue). The mean and S.D. of separability over all parcels are shown in gray.

When raw pairwise distances were used, almost all parcels (613 out of 758 parcels) exhibited significant separability$\Delta_{w}^{BW}$. When residual pairwise distances were considered, ninety-three parcels retained significant$\Delta_{w}^{BW}$(left margin ofFig. 4B). In 28 of these 93 parcels, between-community separability was higher ($t^{BW}>0$) and in the remaining parcels, it was lower ($t^{BW}<0$).

This disparity between raw and residual distances shows that community structure is confounded by temporal auto-correlation to a considerable degree. This is also evident from strong dependence of$t^{BW}$on the range of temporal latencies$\tau_{sig}$(Fig. 4B). When only latency ranges including shorter latencies are considered ($\tau_{sig}\leq 5$), many more parcels are consistently significant than when ranges excluding shorter latencies are also considered ($\tau_{sig}>15$). Applying the strictest criterion and considering only parcels with significant$\Delta_{w}^{BW}$(FDR correctedp<0.05) for all latency bounds$\tau_{LB}\in \left\{1,\ldots ,30\right\}$($\tau_{sig}=30$), we obtained 27 parcels that we considered*‘community sensitive’*. These parcels are listed inAppendix Table A1and illustrated inFigure 4C to Dand inSupplementary Figure S1.

The above analysis yielded interpretable results for strongly-structured presentation sequences, where every object can be objectively assigned to one particular community. When the analysis was repeated for unstructured presentation sequences (by counterfactually assuming a structured sequence and assigning communities accordingly), no systematic results were obtained, as shown inSupplementary Figure S3. Specifically, apparent community sensitivity is observed only when uncorrected distances over low-latency ranges are considered. Correcting for temporal correlations eliminates this spurious sensitivity. The static matrix of average pairwise distances provides an instructive baseline for spurious ‘sensitivity’ that is entirely due to temporal correlations. Apart from very short latencies, the results from this matrix are comparable to results from unstructured sequences, for both*positively*and*negatively*community-sensitive parcels temporal correlations (Supplementary Fig. S3B, C). Results for structured sequences are dramatically different (both higher and lower), corroborating the validity of our analysis of community sensitivity.

Fourteen community-sensitive parcels with*higher*separability of between-community pairs ($\Delta^{BW}>0$) were located in bilateral occipital regions and in ventral occipitotemporal regions of the right hemisphere (visual cortex, lateral occipital cortex, fusiform and lingual gyrus, anterior inferior temporal cortex, as well as intraparietal cortex and middle frontal cortex;Fig. 4C;Appendix Table A1). Eleven of these parcels were also identity-selective. Thirteen other parcels exhibited significantly*lower*separability of between-community pairs ($\Delta^{BW}<0$) and were located in the superior temporal cortex, supramarginal gyrus, insula, operculum, medial frontal cortex, and the frontal pole (Fig. 4C;Appendix Table A1). In this latter group, 12 parcels were not identity-selective.

**Fig. 5.**
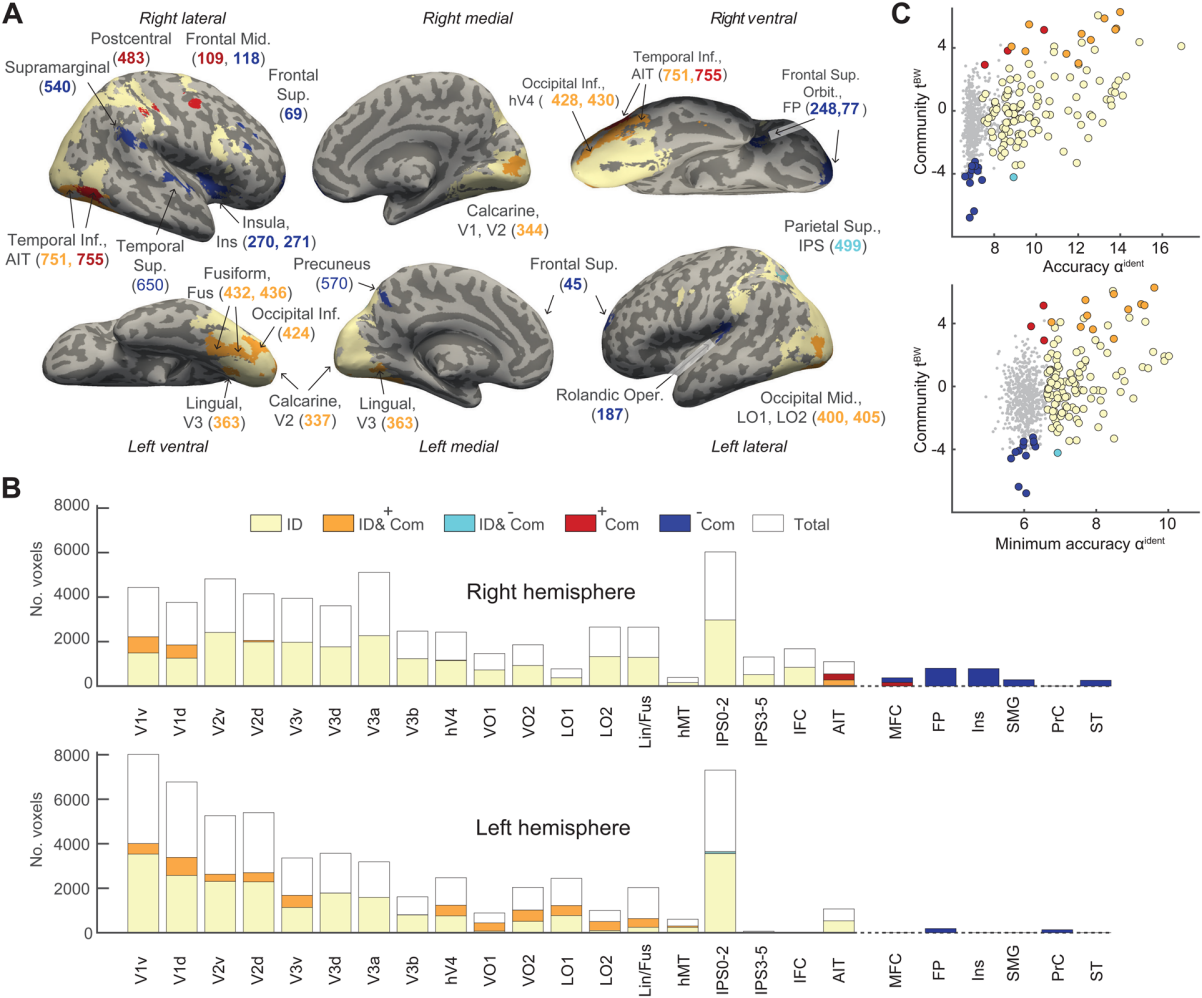
Comparison of selectivity for object identity and sensitivity to community structure. (A) Anatomical distribution of 142 parcels that are identity-selective, community-sensitive, or both. 11 parcels (orange) are both identity-selective and*positively*community-sensitive, while 1 parcel (cyan) combines identity-selectivity with*negative*community-sensitivity. 15 parcels are exclusively community-sensitive, 3 parcels*positively*(red) and 12 parcels*negatively*(blue). The remaining 112 parcels (yellow) are exclusively identity-selective. (B) Share of identity and community representation in 142 parcels with significant representation, assigned to 29 topographical regions, as defined byL. Wang et al. (2015). In the right hemisphere, two parcels (428 and 430) are missing because they could not be assigned to any topographical regions. Coloring corresponds to (A) and indicates the fraction of voxels from parcels with different selectivity. Visual cortex (V1-hV4), ventral occipital cortex (VO), lateral occipital cortex (LO), lingual and fusiform gyri (LIN/FS), medial temporal areas (MST, hMT), intraparietal sulcus (IPS), superior parietal lobule (SPL), anterior inferior temporal cortex (AIT), insula and supramarginal gyrus (INS/SMG), inferior frontal cortex (IFC), medial frontal cortex (MFC), and frontal pole (FP). (C) Quantitative comparison of selectivity for identity and sensitivity for community over all parcels. Identity-selectivity is quantified either by average classification accuracy$\alpha^{identity}$(top) or by the minimum statistic of classification accuracy (bottom). Community-sensitivity is measured by positive or negative values of$t^{BW}$. Significantly sensitive parcels are represented by colored disks, and non-sensitive parcels by grey dots. Coloring corresponds to (A).

The respective cortical distributions of the representations of object identity and community membership are compared and illustrated inFigure 5. The criterion for community-sensitivity was a significantly positive or negative*t*-score value$t^{BW}$, whereas the criterion for identity-selectivity was a significantly positive minimum statistic of classification accuracy$\alpha_{min}$(for details, seeKakaei & Braun, 2024).Figure 5Cshows average classification accuracy$\alpha^{identity}$as well as$\alpha_{min}$. Coloring indicates whether parcels combined identity-selectivity with*positive*community-sensitivity (11 parcels, orange) or*negative*community-sensitivity (1 parcel, cyan), or whether parcels were either exclusively community-sensitive (3 parcels*positively*in red, 12 parcels*negatively*in blue) or exclusively identity-selective (112 parcels, yellow). Of the 124 identity-selective parcels, 12 parcels (approximately 10%) were additionally community-sensitive. Jointly selective/sensitive parcels were most common in the mid-level visual cortex (ventral occipital cortex, lingual and fusiform gyrus) and somewhat less common in the early visual cortex (V1, V2, V3, hV4). Jointly selective/sensitive parcels were largely absent from high-level visual areas in the parietal and frontal cortex (inferior parietal sulcus, superior parietal lobule, insula, inferior and medial frontal cortex), but were present in the anterior inferior temporal cortex. The one negatively community-sensitive parcel in the intraparietal sulcus appeared to be an exception. In summary, jointly selective/sensitive parcels were present at all levels of the ventral visual pathway.

Note that the comparison of community- and identity-selectivity was skewed by disparate statistical power. The assessment of community sensitivity was based on approximately225times fewer observed response distances than the assessment of identity-selectivity (seeSection 2), so statistical sensitivity was expected to be approximately15times lower. Accordingly, if community-sensitivity was detected in only a fraction of identity-selective parcels, this could, in part, have been due to this disparity in statistical power.

Nominally non-identity-selective parcels with*positive*community-sensitive were located in the anterior inferior temporal cortex and in the medial frontal cortex. As seen in the top panel ofFigure 5C, the average classification accuracy$\alpha^{identity}$of these 3 parcels was comparable to other identity-selective parcels. However, these parcels just missed the minimum statistics criterion for significance, as seen in the bottom panel. It seems possible that community-sensitivity degraded identity-selectivity in these parcels, in the sense that reduced response distances within a community might also have reduced distances between the different objects of this community.

Non-identity-selective parcels with*negative*community-sensitivity were located in the insula, the medial frontal cortex, and at the frontal pole. These 11 parcels exhibited no trace of identity-selectivity in terms of either the observer average or the minimum statistics. Negative sensitivity implies that responses to objects from different communities were more similar than responses to objects from the same community. As discussed below, it seems possible that the responses in these areas placed particular emphasis on ‘linking objects’, thereby highlighting the ‘novelty’ or ‘surprise’ associated with the transition to another community and the appearance of unexpected objects.

### 3.3 Representation of object pairs

Structured presentation sequences consist of different types of object pairs, such as adjacent and non-adjacent pairs, or ‘linking’ pairs (between different communities) and ‘internal’ pairs (within the same community). Thus, it was natural to wonder whether different types of object pairs might have contributed differentially to our average measure,$t^{BW}$, for “community sensitivity”?

To address this question, we compared the statistical significance of the signed difference in response distances between and within communities for all object pairs,$t^{BW}$, and for specific types of object pairs: non-adjacent objects in different communities (DN), non-adjacent objects in the same community (SN), adjacent objects in different communities (DA), and adjacent objects in the same community (SA). The results are shown inFigure 6. The separability measure$t^{SA}$was negatively correlated with$t^{BW}$(ρ=−0.91,p<0.01), whereas the measure$t^{DN}$was positively correlated with$t^{BW}$(ρ=0.93,p≪0.01). The separability measures$t^{DA}$and$t^{SN}$were also negative correlated with$t^{BW}$, though much less strongly (ρ=−0.15,p<0.01andρ=−0.19,p<0.01; respectively). These results were robust and held for all lower temporal bounds$\tau_{LB}\leq 30$, except for the correlation between$t^{BW}$and$t^{DA}$, which held only for$\tau_{LB}\leq 28$.

**Fig. 6.**
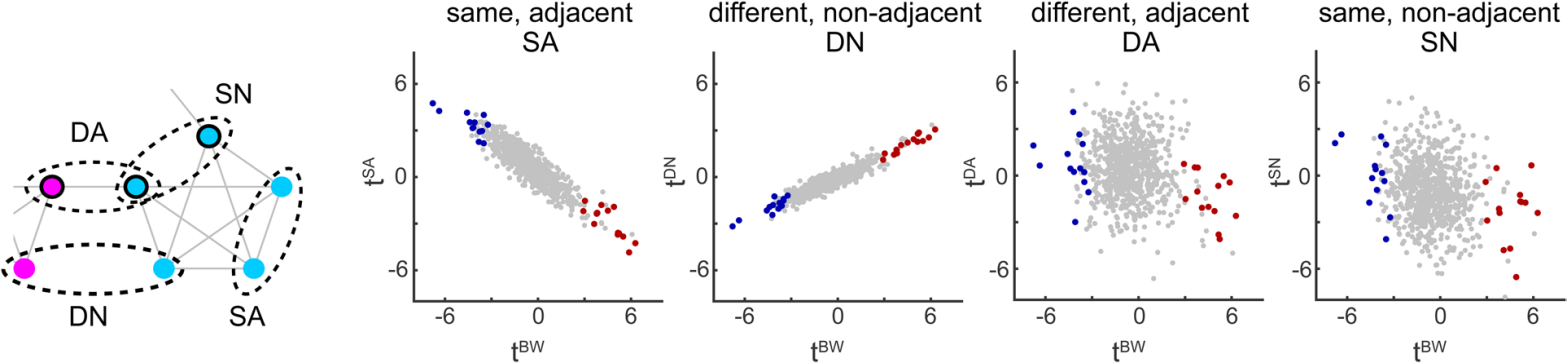
Neural representation of different types of object pairs. Pairs of recurring objects may be in the same (*S*) or different (*D*) communities, and may occupy adjacent (*A*) or non-adjacent (*N*) positions on the path. Differential separability of between- and within-community pairs (measured by t-score value$t^{BW}$) is compared to separability$t^{SA}$of adjacent objects in the same community (left),$t^{DN}$of non-adjacent objects in different communities (middle left),$t^{DA}$of adjacent objects in different communities (middle right), and$t^{SN}$of non-adjacent objects in the same community (right), for all 758 parcels. Community-sensitive parcels are shown in red or blue (as inFig. 4C).

These results show that the representation of community structure (indexed by$t^{BW}$) includes a reduced separation ofSApairs (indexed by$t^{SA}$), as well as an increased separation ofDNpairs (indexed by$t^{DN}$). Recall thatSA(andDA) pairs occur in presentation sequences (with probability1/60), whereasSN(andDN) pairs never occur. The selective modulation of representational distance for one of the two adjacent (and therefore occurring) pairs appears to be a correlate of temporal community structure. The same can be said for the selective modulation of representational distance for one of the two non-adjacent (and therefore non-occurring) pairs. Furthermore, the correlation between community representation and separation ofSAandDNpairs is evident not only in the few parcels meeting the statistical threshold for community sensitivity (red and blue dots inFig. 6), but also in all other parcels as well (grey dots inFig. 6). Thus, reduced separation ofSApairs and increased separation ofDNpairs appear to be a general feature of the cortical representation of community structure.

The results described above depend critically on the correction for temporal correlations (Supplementary Fig. S4). Without this correction, the$t^{BW}$measure for between-community separation is dominated by the influence of short-latency pairs. When shorter latencies are excluded and$\tau_{LB}\geq 5$, the correction ceases to make a difference. This underlines again that correcting for average temporal correlations is key to establishing representations of community structure.

### 3.4 Representational space

A previous study with structured sequences (A. C. Schapiro et al., 2013) reported that within-community distances are typically smaller than between-community distances and illustrated this finding with multidimensional scaling. We sought to replicate this by visualizing the relative proximity of different types of object pairs. To obtain interpretable results, we employed a permutation procedure that allowed us to average proximity matrices over observers (seeSection 2for details). The resulting arrangements exhibited a three-fold rotational symmetry that was owed to this permutation procedure and therefore was artificial.

For the 14*positively*community-sensitive parcels, the relative proximity of different object pairs is illustrated inFigure 7. In all cases except one, objects were clustered by community (i.e., spaced more closely within than between communities), with Temporal-Inf-R-10 providing the most extreme example. Additionally, ‘linking’ objects tended to be positioned differently than internal objects, in all but two cases closer to each other (and to the center) (Calcarine-L-9, Calcarine-R-5, Lingual-L-1, Occiptal-Mid-L-4, Occiptial-Mid-L-9, Occipital-Inf-L-2, Fusiform-L-2, Fusiform-L-6, Postcentral-R-11, Temporal-Inf-R-10). Exceptions were Frontal-Mid-R-7, where only internal objects clustered by community, and Occipital-Inf-R2/4, where linking objects were more distant from each other. As these illustrations show only relative distances,Supplementary Figure S6Aprovides absolute response distances in terms of the average and standard error over parcels, separately for internal objects and linking objects, as well as within and between communities. Response distances of internal objects within the same community correspond to the grand average over all object pairs, whereas distances between different communities were significantly larger. Additionally, distances between linking and internal (or linking) objects within the same community were significantly smaller. Thus, both clustering by community and relative proximity of linking objects was statistically significant. On average, this corresponded to the possibility shown schematically inFigure 3A.

**Fig. 7.**
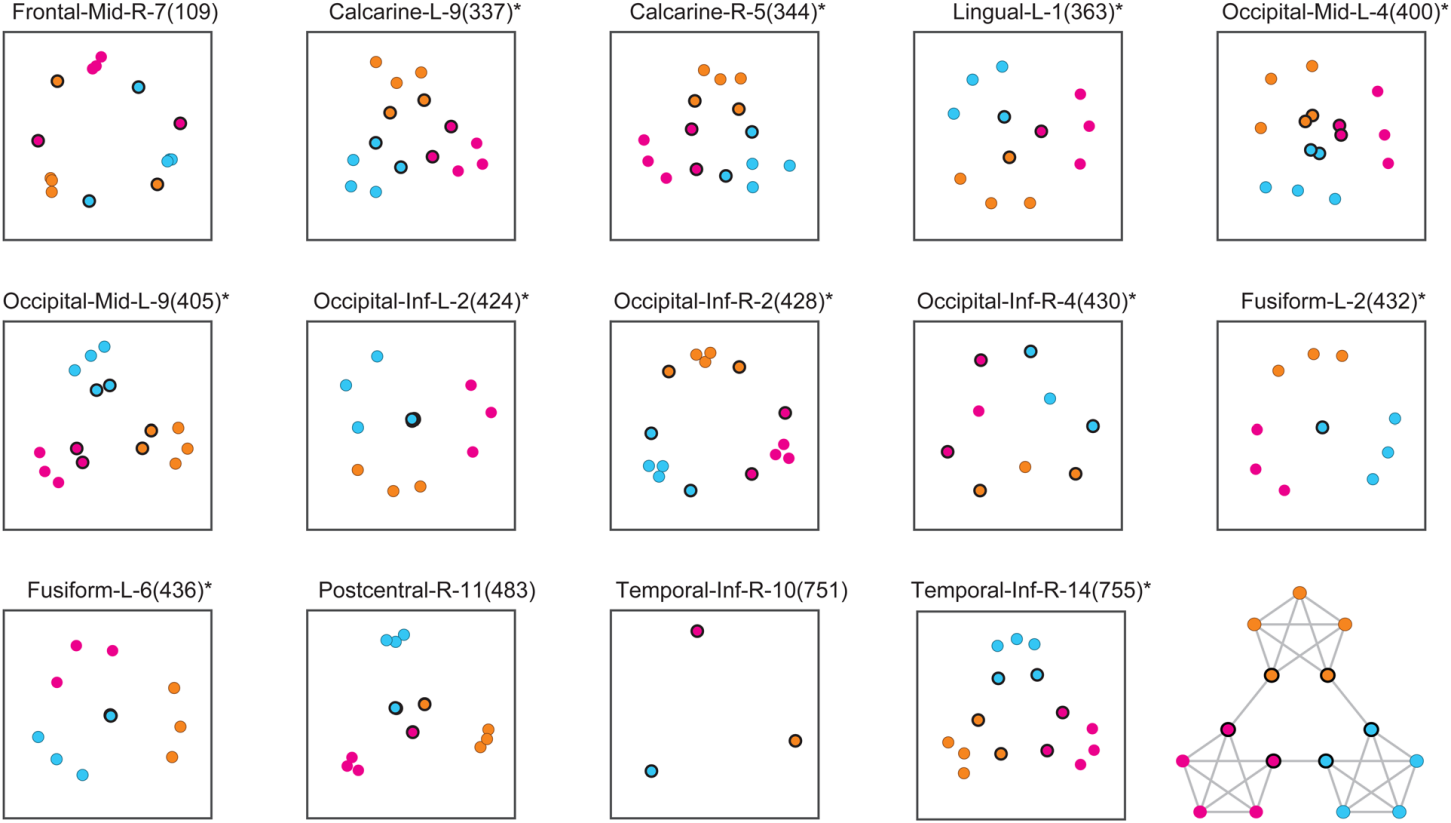
Representation of temporal community structure in positively sensitive parcels. Multidimensional reduction of the pairwise distance matrix averaged over path permutations and over observers. Communities are distinguished by color and linking objects by a black outline, as indicated by the path diagram (inset). Fourteen parcels exhibited higher separability between communities than within communities ($t^{BW}>0$). Identity-selective parcels are marked with⋆.

Results for the 13*negatively*community-sensitive parcels are shown inFigure 8. The clustering of internal objects (Frontal-Sup-L-12, Frontal-Sup-Orb-R-3, Frontal-Mid-R-16, Frontal-Med-Orb-R-3, Parietal-Sup-L-8, and Temporal-Sup-R-6) was variable but, when averaged over parcels, internal objects were more distant within than between communities (Supplementary Fig. S6A). Specifically, within the same community, response distances of internal objects to other internal objects (or linking objects) were significantly larger than the grand average over all object pairs, whereas between communities response distances were smaller. On average, this corresponded to the possibility shown schematically inFigure 3B. In six parcels, all linking objects were distant from each other (and from the center), suggesting that the representation in these parcels individuated different transitions between communities (Frontal-Sup-R-19, Frontal-Sup-Orb-R-3, Insula-R-6, Parietal-Sup-L-8, Precuneus-L-12, Putamen-R-5). However, in seven other parcels, linking objects were separated less well than internal objects (Frontal-Sup-L-12, Frontal-Mid-R-16, Rolandic-Oper-L-2, Frontal-Med-Orb-R-3, Insula-R-5, SupraMarginal-R-4, Temporal-Sup-R-6), suggesting that the representation conflated different transitions between communities.

**Fig. 8.**
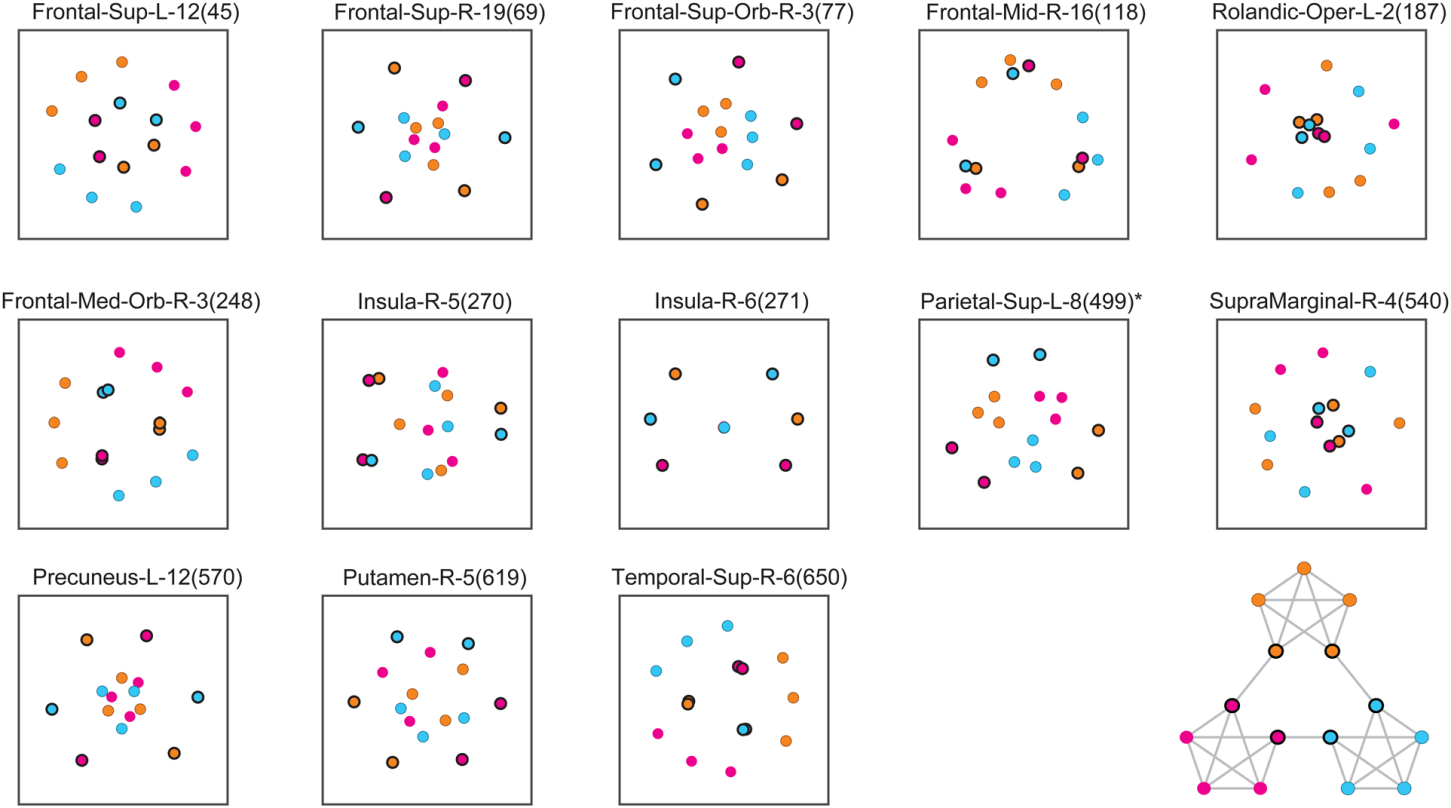
Representation of temporal community structure in negatively sensitive parcels. Multidimensional reduction of the pair-wise distance matrix averaged over path permutations and observers. Communities are distinguished by color and linking objects by a black outline, as indicated by the path diagram (inset). Thirteen parcels exhibited lower separability between communities than within communities ($t^{BW}<0$). Identity-selective parcels are marked with⋆.

It is instructive to also compare parcels that were not classified as either identity-selective or community-sensitive. Results for 15 randomly chosen ‘non-selective’ parcels are shown inSupplementary Figure S5. Perhaps not surprisingly, the results were quite heterogeneous and few differences reached statistical significance when averaged over parcels (Supplementary Fig. S6A). However, in several individual parcels, clustering by communities and/or prominent representation of linking objects was evident.

As a final control, we analyzed average pairwise distances in the responses obtained with unstructured sequences. Here, we failed to observe significant deviations from the grand average distance, either for internal and linking objects or within and between communities (Supplementary Fig. S6B). This corroborates that the results obtained with structured sequences were due to the presence of temporal structure and/or temporal communities.

## 4 Discussion

We investigated incidental and automatic learning of regularities and dependencies without explicit behavioral task (Aslin, 2017;Fiser & Lengyel, 2022;Perruchet, 2019;Perruchet & Pacton, 2006;Saffran & Kirkham, 2018;A. Schapiro & Turk-Browne, 2015). Our aim was to compare the cortical basis of concurrent learning of statistical structure with two timescales, namely, explicit learning to recognize complex objects presented for~3 s (Cox et al., 2005;Tian & Grill-Spector, 2015;Wallis & Bülthoff, 2001) and implicit learning of task-irrelevant contingencies in the sequence of object presentations (“temporal communities” lasting~30 s) (Fiser & Aslin, 2002;Kakaei et al., 2021;Miyashita, 1988;Sáringer et al., 2022;Turk-Browne et al., 2005,^2009^). Our results show that cortical representations of both object identity and temporal community structure coexist in large parts of the ventral occipitotemporal cortex.

Previous studies have localized view-invariant object representations in inferior temporal cortex (IT) and lateral occipital complex (LOC) (Grill-Spector et al., 2001;Sáry et al., 1993;Van Meel & Op de Beeck, 2020). Single-unit responses in IT of non-human primates reflect the intrinsic contingencies of an invariant representation and correlate closely with recognition performance (Jia et al., 2021;Li & DiCarlo, 2008,^2010^,^2012^). Human fMRI show differential adaptation in IT for congruent and incongruent shapes (Van Meel & Op de Beeck, 2018). In addition, evidence for view-invariant representations has been reported in primary-visual cortex (Eger et al., 2008), at more anterior sites such as fusiform gyrus, and ventral occipito-temporal cortex (Brants et al., 2016;Visconti di Oleggio Castello et al., 2021), as well as in several areas of the dorsal pathway (Freud et al., 2017;Jeong & Xu, 2016;Konen & Kastner, 2008;Poirier et al., 2006;Visconti di Oleggio Castello et al., 2021).

Our results confirm and extend these previous findings on cortical regions with view-invariant object representations, as described in our companion study (Kakaei & Braun, 2024). In brief, we established cross-validated multivariate representations of object identity for smallish ‘functional parcels’ ($\sim 1.7cm^{3}$cortex volume) defined previously by a functional parcellation (MD758;Dornas & Braun, 2018). Parcels in which significant identity information was prevalent (Allefeld et al., 2016) were located in both the ventral and dorsal visual pathways, beginning with early visual areas (V1-hV4), extending to more anterior parts of ventral occipitotemporal cortex into anterior inferior temporal cortex, as well as to anterior inferior frontal cortex (Kakaei & Braun, 2024).

Our motivation to compare cortical representations of object shape and temporal object sequence derived from classical studies of object recognition in non-human primates (Erickson & Desimone, 1999;Miyashita, 1988). These studies had shown that the responsiveness of single neurons in the inferiotemporal cortex developed selectivity not only for the identity but also for the presentation order of objects, provided that animals had consistently viewed these visual objects in the same sequential order. As this order was irrelevant to the animal’s task, the development of a neural representation for sequential order constituted a prototypical instance of incidental or implicit learning.

In extensive subsequent work with “paired-associate tasks”, the sequential order of objects was made task-relevant so that learning of temporal associations became explicit. Over the course of training, the prevalence of pair-encoding neurons was found to increase in anterior parts of inferiotemporal cortex IT (Hirabayashi & Miyashita, 2014;Messinger et al., 2001;Naya et al., 2001,^2003^). Additionally, neurons in IT were found to encode “object-general semantic value” in the sense of identifying whether a particular object was “familiar” or “novel” (Tamura et al., 2017). Here, we investigated the possibility that such “object-general” information could extend to membership in a “temporal community” of objects.

Previous behavioral studies have shown that humans can implicitly learn spatiotemporal associations between objects and use these regularities to enhance their cognitive performance. Observers can automatically capture spatial (Fiser & Aslin, 2001) and temporal (Fiser & Aslin, 2002;Turk-Browne et al., 2008) regularities as both joint and conditional probabilities of stimuli co-occurrence. This surpasses simple object-object associations and extends to higher-order association probabilities, over multiple objects. Even when the conditional probability between object pairs is uniform and thus uninformative of the underlying association between objects, humans are sensitive to higher-order regularities (higher-moments of conditional probability distribution) (Kahn et al., 2018;Kakaei et al., 2021;Karuza, Kahn, et al., 2017;A. C. Schapiro et al., 2013). This capability for incidental learning of complex regularities can facilitate performance in various domains, including language (e.g.,Saffran et al., 1996), motor (e.g.,Hunt & Aslin, 2001), spatial attention (e.g.,Chun & Jiang, 1998;Jiang & Wagner, 2004), and object recognition learning (e.g.,Kakaei et al., 2021).

The literature on implicit or explicit learning of temporal associations shows that both domain-specific and domain-general brain regions can be involved (for reviews, seeBatterink et al., 2019;Fiser & Lengyel, 2022). Neural correlates of statistical learning are evident in early domain-specific sensory areas where spatiotemporal regularities are first extracted, to mid-level sensory areas where these representations are supposedly integrated. In the visual domain, spatiotemporal regularities emerge in lateral and ventral occipito-temporal and parieto-occipital regions in humans (Henin et al., 2021;Karuza, Emberson, et al., 2017;Rosenthal et al., 2016;Turk-Browne et al., 2009) and are observed in inferiotemporal and anterior inferiotemporal regions in non-human primates (Kaposvari et al., 2018;Meyer et al., 2014;Miyashita, 1988;Sakai & Miyashita, 1991). More abstract and generalized representations of temporal associations have been reported in more downstream, domain-general areas such as medial temporal lobe, striatum and frontal regions. Moreover, the majority of studies point to an essential role of the medial temporal lobe (MTL), particularly the hippocampus, in statistical learning (Hindy et al., 2016;Hsieh et al., 2014;A. Schapiro & Turk-Browne, 2015;A. C. Schapiro et al., 2012,^2013^,^2016^;Schendan et al., 2003;Turk-Browne et al., 2009,^2010^). This is particularly true when sequences are repeated and when ordinal knowledge is of particular interest to observers (for reviews, seeDavachi & DuBrow, 2015;Eichenbaum et al., 2016). MTL seems to be engaged in statistical learning that occurs early in the learning process but seems to disengage as learning progresses, particularly after consolidation. Concurrently, the encoding of statistical knowledge seems to transfer from MTL to the striatal-frontal network (Batterink et al., 2019;Durrant et al., 2013). Higher cortical regions in insular cortex and prefrontal cortex (PFC), including inferior frontal gyrus (IFG) and medial prefrontal cortex (mPFC), also show sensitivity to statistical regularities, particularly when the complexity increases (Giorgio et al., 2018;Henin et al., 2021;Karlaftis et al., 2019;Kourtzi & Welchman, 2019;A. C. Schapiro et al., 2013;R. Wang et al., 2017).

Here, we adapted the paradigm ofA. C. Schapiro et al. (2013)and used object sequences with higher-order “temporal community structure”. In such sequences, pair probabilities are uniform in that every object is succeeded by one of four other objects with equal probability. This avoids the novelty/surprise effects that would arise if some object transitions were more common or rare than others. We term sequences with temporal communities “strongly structured”, to distinguish them from “unstructured” pseudo-random sequences where every object can be succeeded by any other object (Kakaei et al., 2021).

We studied cortical representations with a “representational similarity analysis” (RSA) approach, which relies on comparing pairwise distances between multivariate BOLD responses to different objects. A difficulty with this approach is that multivariate BOLD patterns are known to be significantly autocorrelated over 10 s of seconds (Alink et al., 2015;Henriksson et al., 2015), in part due to hemodynamic effects (Friston et al., 1994;Zarahn et al., 1997). Accordingly, it was essential to distinguish between response similarity due to genuine “temporal community” effects and response similarity due to mere temporal proximity (i.e., systematically shorter latencies between objects in the same community) (Cai et al., 2019;Gilron et al., 2016).

We took two measures to control for this confound and to distinguish between community and latency effects. First, we computed and analyzed ‘residual distances’ by subtracting from each observed distance at a certain latency the*average*distance at that latency (seeSection 2.6.1). Second, we assessed consistency by analyzing and comparing distances in different latency ranges, for example including or excluding short latencies. These measures turned out to be essential, as nearly the entire brain would have spuriously appeared to be ‘community-sensitivity’ without them. They also proved effective, as they revealed ‘community-sensitivity’ only in multivariate BOLD responses to “strongly-structured” sequences and not in responses to “unstructured” sequences. Accordingly, we are confident that these measures identify genuine cortical representations of “temporal community”.

Our analysis of multivariate BOLD responses in 758 ‘functional parcels’ revealed two functionally and anatomically distinct kinds of ‘community-sensitivity’ (see alsoFig. 3). The first kind—termed*positively-sensitive*—showed*greater*similarity of responses within communities than between communities and was observed mostly in domain-specific, visual brain regions. The second kind—termed*negatively-sensitive*—exhibited*lesser*similarity of responses within communities and was observed mostly in domain-general areas. We now discuss these two groups in more detail.

Positively community-sensitive parcels—where response distances were*smaller*for objects within than between communities—were located almost exclusively in ventral occipitotemporal cortex, with seven parcels in the left hemisphere (Calcarine-337, Occipital-Inf-424, Occipital-Mid-400 and -405, Fusiform-432 and -436, Lingual-363) and five parcels in the right hemisphere (Calcarine-344, Occipital-Inf-428 and -430, Temporal-Inf-751 and -755). Thus, community-sensitive parcels spanned the range of ventral occipitotemporal cortex that also contained parcels selective for object identity. Almost all positively community-sensitive parcels also exhibited significant identity-selectivity. Although the pattern of relative response distances was somewhat heterogeneous (Fig. 7), some significant trends emerged: response distances between ‘internal’ objects were above average between communities, whereas distances between ‘linking’ objects were below average both within and between communities (Supplementary Fig. S6).

While positively community-sensitive parcels comprised only a small fraction of identity-selective parcels (11 of 124 parcels), this disparity may exaggerate the true situation. As our paradigm was considerably less sensitive for community than for identity, a number of ‘false negatives’ was only to be expected. If the respective statistical sensitivities had been comparable, the overlap between the two groups might well have been larger.

These results are consistent with earlier findings that early and mid-level visual areas are sensitive to temporal regularities and can flexibly alter their activity pattern to represent the temporal context (Henin et al., 2021;Karuza, Emberson, et al., 2017;Rosenthal et al., 2016;Turk-Browne et al., 2009). These are also consistent with the classical observation that representations of temporal association develop conjointly with representations of object identity (Erickson & Desimone, 1999;Miyashita, 1988).

In contrast to numerous earlier studies (Hindy et al., 2016;Hsieh et al., 2014;A. Schapiro & Turk-Browne, 2015;A. C. Schapiro et al., 2012,^2013^,^2016^;Schendan et al., 2003;Turk-Browne et al., 2009,^2010^), we failed to observe positive community-sensitivity in the medial temporal lobe (MTL). We do not consider this a contradiction, as our analysis did not focus on MTL and our parcellation included only six parcels in this region (2×Hippocampus, 2×Perirhinal, 2×Amygdala). Moreover, MTL is thought to engage early in the learning process and the memory engram is thought be transferred to the striatum after consolidation. As our observations spanned multiple days, memory consolidation could have occurred already after the first session, which could also have explained our failure to observe any community-sensitivity in the MTL. Interestingly, we did observe such sensitivity in one parcel of the putamen.

Negatively community-sensitive parcels were located in domain-general cortex, including the temporal cortex (Temporal-Sup-R-650), parietal cortex (Parietal-Sup-L-499, Supramarginal-R-540, Precuneus-L570), superior frontal cortex (Sup-Frontal-L-45 and -R-69, Sup-Frontal-Orbit-77, Sup-Frontal-Med-R-248), and middle and inferior frontal cortex (Mid-Frontal-R-118, Insula-R-270 and -271, and Rolandic-Oper-L-187). Apart from Parietal-Sup-L499, none of these parcels exhibited a significant representation of object identity, further strengthening the dissociation between*negative*and*positive*community representations.

These findings are consistent with previous reports that implicit learning paradigms can engage parieto-frontal, fronto-striatal, and/or ventral attention networks (Batterink et al., 2019). More generally, prefrontal cortex (PFC) is thought to reflect higher-order statistics of event (Henin et al., 2021) and decision strategies adopted by observers (Giorgio et al., 2018;Karlaftis et al., 2019;Kourtzi & Welchman, 2019;R. Wang et al., 2017). Orbitofrontal cortex (OFC) is thought to be engaged when more abstract representations or ‘cognitive maps’ are required (Behrens et al., 2018;Christophel et al., 2017;Knudsen & Wallis, 2022;Rusu & Pennartz, 2020;Schuck et al., 2016;Wilson et al., 2014). Insula and inferior frontal gyrus are thought to be engaged by working memory tasks, especially under conditions of high load (Rottschy et al., 2012), and to contribute to goal-directed behavior by interacting with the medial temporal lobe hippocampus (Rusu & Pennartz, 2020). Moreover, when objects are viewed in temporally structured sequences, responses in insula and inferior frontal gyrus are suppressed for expected objects (Ferrari et al., 2022). Interestingly, this ‘expectation suppression’ arises earlier than in the occipitotemporal visual areas (see alsoWeilnhammer et al., 2021).

The*negative*community-sensitivity observed both here and in previous studies (A. C. Schapiro et al., 2013) is consistent with “context-specific maps” that individuate objects in a given community, without necessarily identifying either the community or objects in other communities. When the context changes, such a map could be reused to individuate objects in the new community. This would be similar to the invariant response patterns in different environments exhibited by grid-cells (Constantinescu et al., 2016;Doeller et al., 2010;Fyhn et al., 2007).

In summary, our results demonstrate incidental learning of temporal associations at all levels of the ventral visual pathway—from the primary visual cortex to the anterior inferior temporal cortex—at the time-scales of both object presentations (seconds) and of temporal contingencies in the object sequence (tens of seconds). This functional overlap suggests that the visual hierarchy develops*convergent representations*(Grill-Spector & Weiner, 2014) that integrate information from a range of time-scales. It seems likely that such convergent representations contribute to context-dependent enhancement of recognition performance. Our findings confirm the classical observation of a conjoint development of representations of object identity and temporal association (Erickson & Desimone, 1999;Miyashita, 1988).

In the domain-general cortex—superior temporal, parietal, frontal, and insular—representations of higher-order temporal context were also evident, but without any stable representations of object identity. Particularly the ‘linking objects’ that separated different temporal communities in structured presentation sequences tended to be represented distinctly. Thus, our finding suggests that both the ventral occipitotemporal cortex and/or domain-general cortex could be in a position to contribute to “structural learning” (Kemp & Tenenbaum, 2008;Tenenbaum et al., 2011) and the development of causal insight and understanding (Lake et al., 2017;Shafto et al., 2011).

## Supplementary Material

Supplementary Material

## Data Availability

Direct linear discriminant analysis and prevalence inference is available on github.com/cognitive-biology/DLDA. MR data will be made available upon request.

## Appendix

**Appendix Table A1.** List of community-selective parcels and their anatomical region.

Region	Parcel		MNI		Community	Identity	Topog.
	No.	X	y	z	$t_{BW}$	α (%)	assign.
Middle frontal	109	48	-3	56	2.9	-	-
Calcarine	337	-12	-99	-5	5.2	13.8	V1d
	344	9	-85	6	5.2	13.8	V1v
Lingual	363	-12	-65	-5	3.8	9.5	V3v
Occipital	400	-38	-86	4	3	12.0	LO2
(middle)	405	-16	-100	1	6.3	14.0	V2d
Occipital	424	-31	-83	-8	4.5	12.6	-
(inferior)	428	36	-85	-7	5.9	13.2	hV4
	430	42	-73	-9	3.6	11.4	hV4
Fusiform	432	-27	-71	-11	4.9	12.2	VO2
	436	-31	-53	-13	4.1	8.8	PHC1
Postcentral	483	51	-26	47	3.8	-	-
Temporal	751	51	-67	-8	5.1	-	AIT
(inferior)	755	46	-53	-11	5.5	9.7	AIT
Superior frontal	45	-23	58	23	-4.1	-	-
	69	21	63	4	-6.4	-	-
Superior frontal	77	23	61	-5	-6.8	-	-
(orbital)							
Middle frontal	118	44	35	34	-3.8	-	-
Rolandic	187	-42	-25	18	-3.2	-	-
operculum							
Superior frontal	248	9	65	-9	-4.6	-	-
(medial orbital)							
Insula	270	39	20	-4	-3.6	-	-
	271	41	7	4	-3.8	-	-
Parietal	499	-28	-69	50	-4.2	8.9	-
(superior)							
Supramarginal	540	56	-44	29	-4.4	-	-
Precuneus	570	-3	-62	46	-3.5	-	-
Putamen	619	26	11	6	-4.2	-	-
Temporal	650	63	-8	5	-3.5	-	-
(superior)							

Numerical parcel ID, geometrical centroid x/y/z in MNI, between-community separability$t^{BW}$, identity classificationα, and topographical assignment, if any.
