# Supplementary material for: Incidental learning of predictive temporal context within cortical representations of visual shape
Source: Imaging Neurosci (Camb). 2024 Aug 30;2:imag-2-00278. doi: 10.1162/imag_a_00278 (PMC12290817; doi:10.1162/imag_a_00278)
Supplement: Supplementary Material [file imag_a_00278-supp.pdf]

Supplementary information: Incidental learning of predictive temporal context within cortical representations of visual shape.

Ehsan Kakaei,[1,2,3*] Jochen Braun,[2,3]

[1]European Structural and Investment Funds Graduate School on Analysis, Imaging, and Modelling of Neuronal and Inflammatory Processes, Otto-von-Guericke University, 39120 Magdeburg, Germany

[2]Institute of Biology, Otto-von-Guericke University, 39120 Magdeburg, Germany

[2]Center for Behavioral Brain Sciences, Otto-von-Guericke University, 39120 Magdeburg, Germany

[*]Correspondence: ehsankakaei91@gmail.com

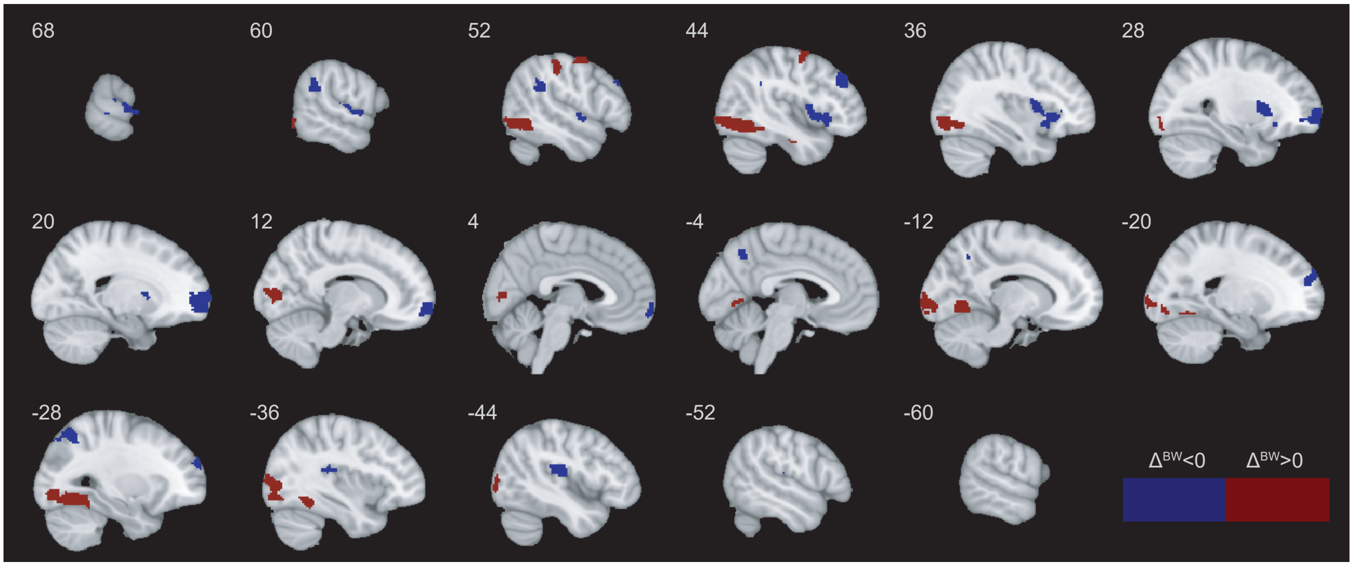

Figure S1: Neural representations of temporal community structure. Positively community-sensitive parcels have significantly higher separability between communities than within (red, $t^{BW} > 0$) and *vice versa* for negatively community-sensitive parcels (blue, $t^{BW} < 0$). Sagittal slices of 8 mm thickness are shown in the range from $X = -60$ to $X = +68$ (MNI).

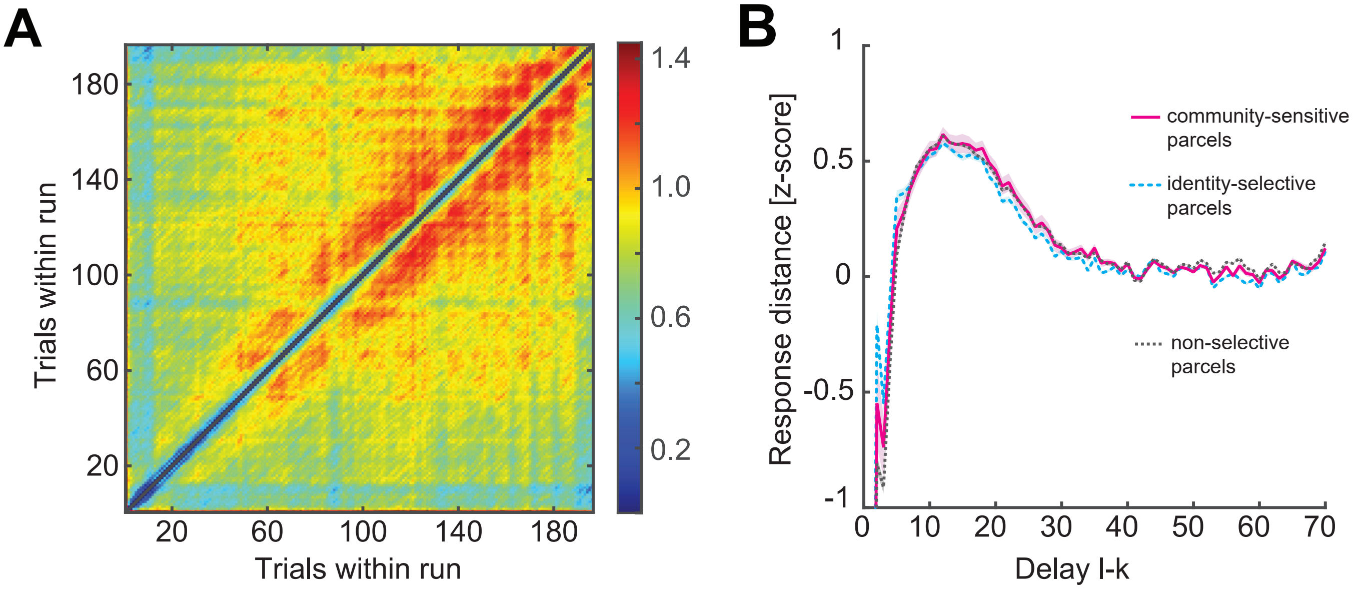

Figure S2: Response distances as a function of delay. Pairwise euclidean distance $d_{w,u,r}(k,l)$ between trials k and l in parcel w, run r, and subject u were averaged over subjects and runs to obtain distance matrix $T_w(k,l) = \langle d_{w,u,r}(k,l) \rangle_{u,r}$ and delay-dependent distance $D_w(\Delta k) = \langle d_{w,u,r}(\Delta k) \rangle_{u,r}$. **A)** Average distance matrix $T(k,l) = \langle T_w(k,l) \rangle_w$ over all parcels. Note that average delay-dependence grows over the course of each run. **B)** Average delay-dependence $T(\Delta k) = \langle D_w(\Delta k) \rangle_w$ for community-sensitive parcels (magenta), identity-selective parcels (cyan), and non-selective parcels (black), in units of z-score (mean $\pm$ S.E.M over parcels). Adapted from **Kakaei2023Gradual**.

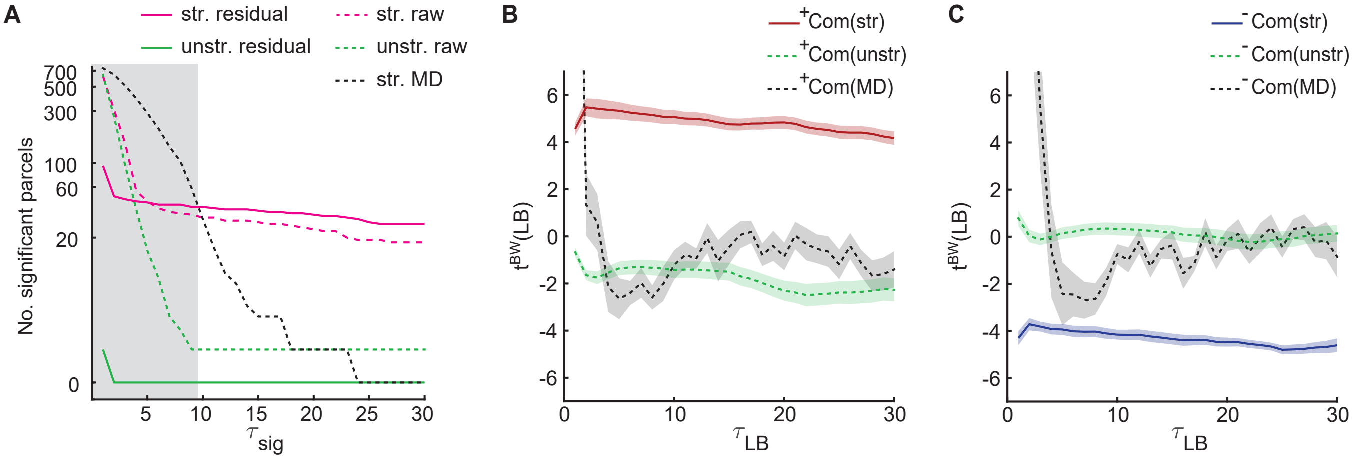

Figure S3: Comparison of controls for community representation. **A)** Apparent community sensitivity, as measured by separability t^{BW} that is consistently significant for all latency ranges with lower bounds $\tau_{LB} \in \{1, 2, \ldots, \tau_{sig}\}$. The number apparently selective of parcels is shown for latency-corrected, residual distances (solid) and for non-latency corrected, raw distances (dashed). Distances were obtained from structured sequences (magenta), from unstructured sequences(green), as well as from static, average distance matrices (black). **B)** Between community separability $t^{BW} \pm S.E.M.$, as function latency lower bound τ^{BW}), for *positively* community selective parcels, based on structured sequences (solid red), unstructured sequences (dashed green), and static average distance matrices (dashed black). **C)** Between community separability t^{BW} calculated for *negatively* community selective parcels, based on structured sequences (solid blue), unstructured sequences (dashed green), and average distance matrices (dashed black).

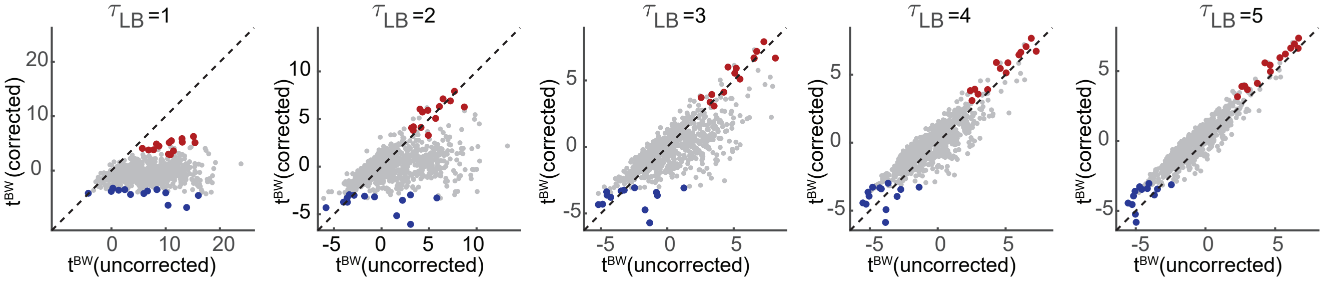

Figure S4: Community sensitivity, latency bound and correction for temporal correlations. Comparison of between-community separability t^{BW} for uncorrected and for corrected distances, as well as for different latency lower bounds τ_{LB}. Parcels classified as *positively* ($\Delta^{BW} > 0$) or *negatively* ($\Delta^{BW} < 0$) community-selective are shown in red and blue, respectively. parcels with higher separability for between-community pairs ($\Delta^{BW} > 0$) and for within-community pairs ($\Delta^{BW} < 0$) are shown in red and blue, respectively. Dashed lines indicate identity.

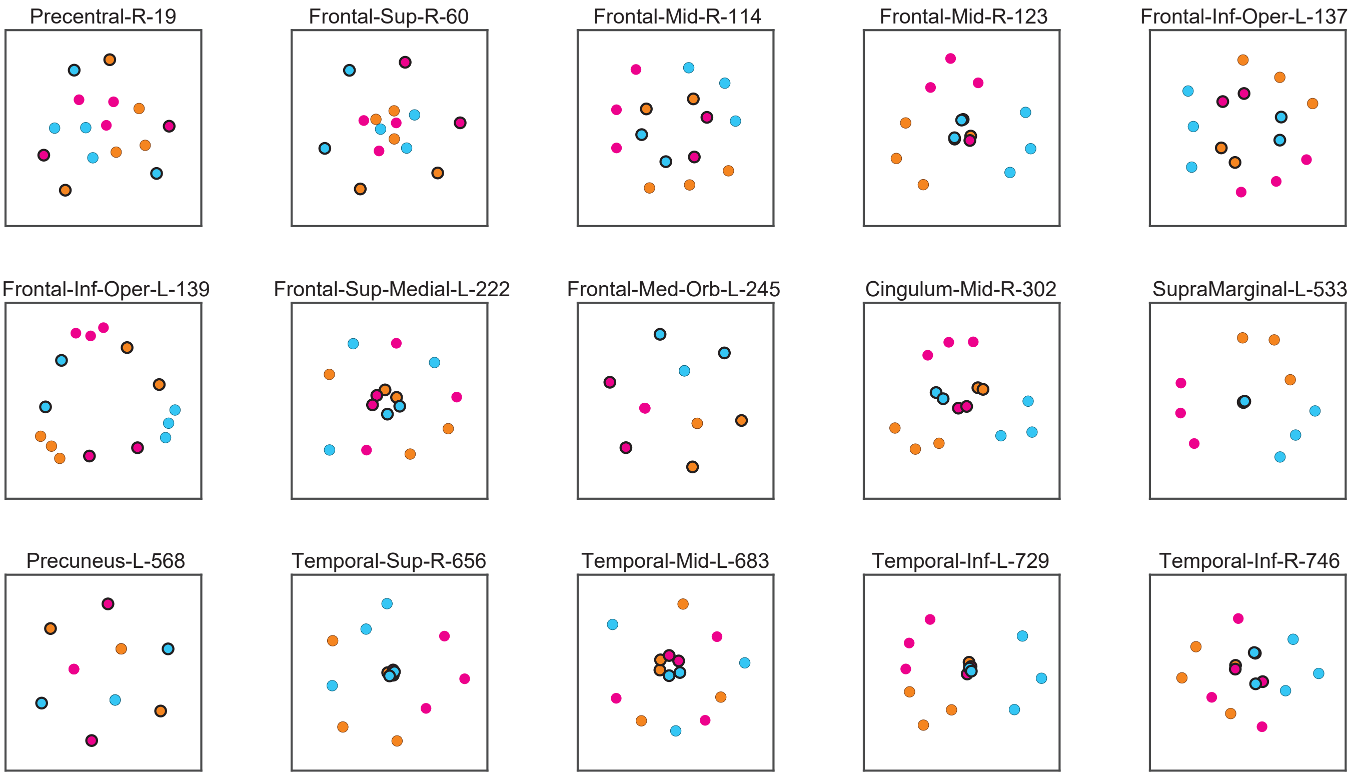

Figure S5: Representation of temporal community structure in other, non-selective parcels. Multidimensional reduction of the pair-wise distance matrix, averaged over path permutations and observers. Communities are distinguished by color and linking objects by a black outline. The fifteen parcels shown were selected randomly from non-indentity- and non-community-selective parcels ($t^{BW} \approx 0$).

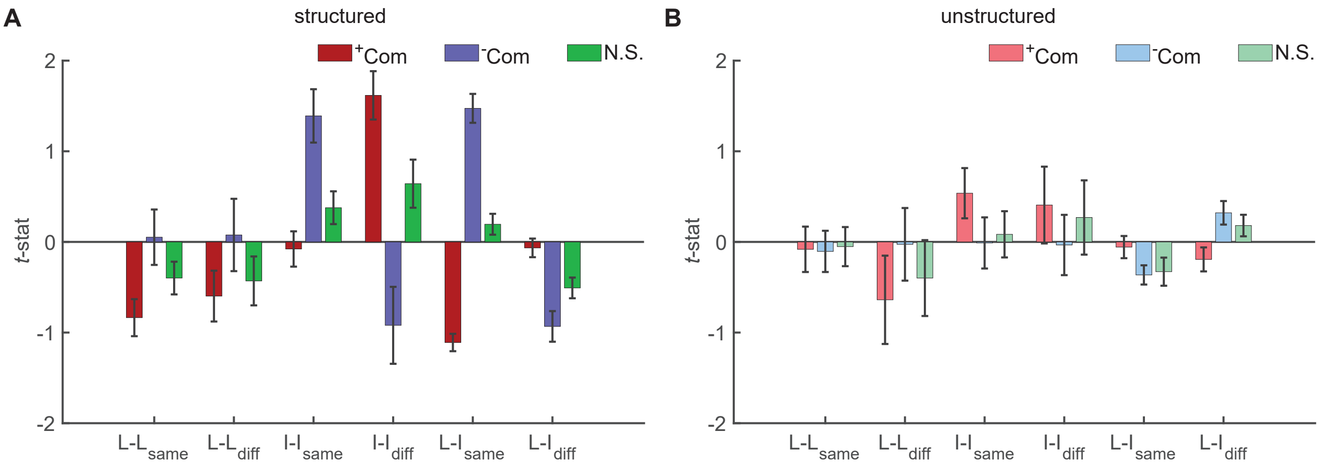

Figure S6: Separability of specific types of object pairs, composed of linking (L) and/or internal (I) objects, within the *same* or in *different* communities, for three different sets of parcels: *positively* community selective parcels (red) (compare **Fig. ??**), *negatively* community selective parcels (blue, compare **Fig. ??**), and randomly chosen, non-selective parcels (green, compare **Fig. S5**). **A)** Separability of object responses in structured sequences. Average separability $t \pm S.E.M$ of each type of object pairs, relative to the ensemble distribution over all types of pairs. Linking objects within communities $(L - L_{same})$, between communities $(L - L_{diff})$, internal objects within $(I - I_{same})$ and between communities $(I - I_{diff})$, as well as linking and internal objects within $(L - I_{same})$ and between communities $(L - I_{diff})$. **B)** Separability of object responses in unstructured sequences. Average separability $t \pm S.E.M$ for each type of object pairs, relative to the ensemble distribution over all types of pairs.

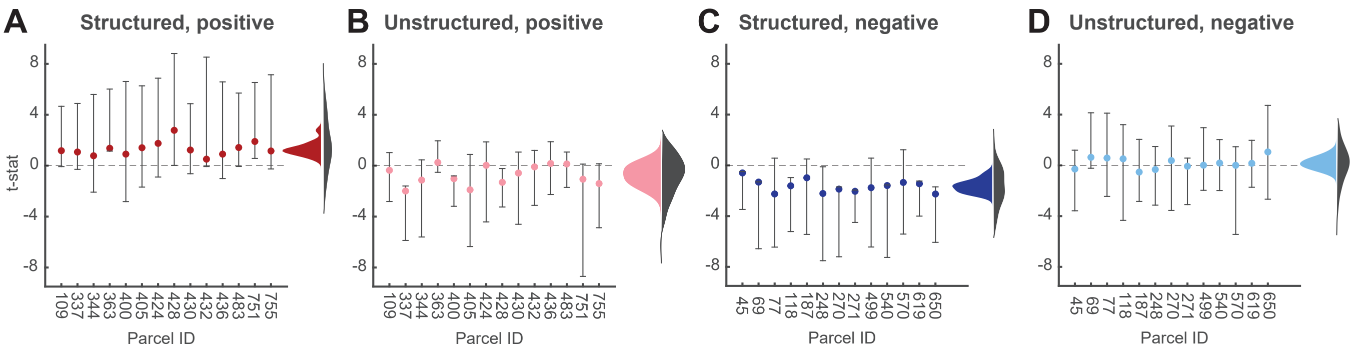

Figure S7: Positive and negative community sensitivity for individual parcels. Median and range over eight observers (colored disks and error bars), fitted distribution of medians (colored distribution at right margin) and of individual observers (black distribution at right margin). **A,C)** Structured presentation sequences show consistent sensitivity. **B,D)** Unstructured sequences do not.

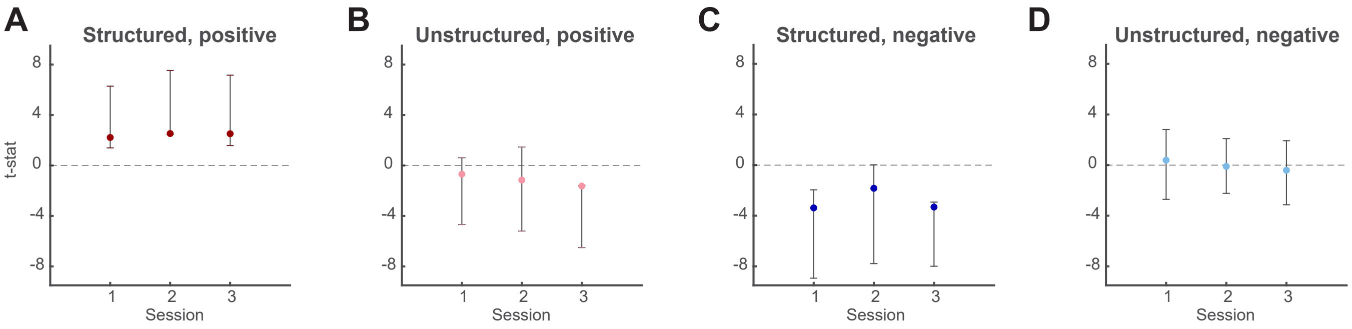

Figure S8: Positive and negative community sensitivity for first, second and third sessions. For each session, the median (colored disk) and the range (error bar) over community-sensitive parcels is shown. **A,C)** Structured presentation sequences show consistent sensitivity. **B,D)** Unstructured sequences do not.

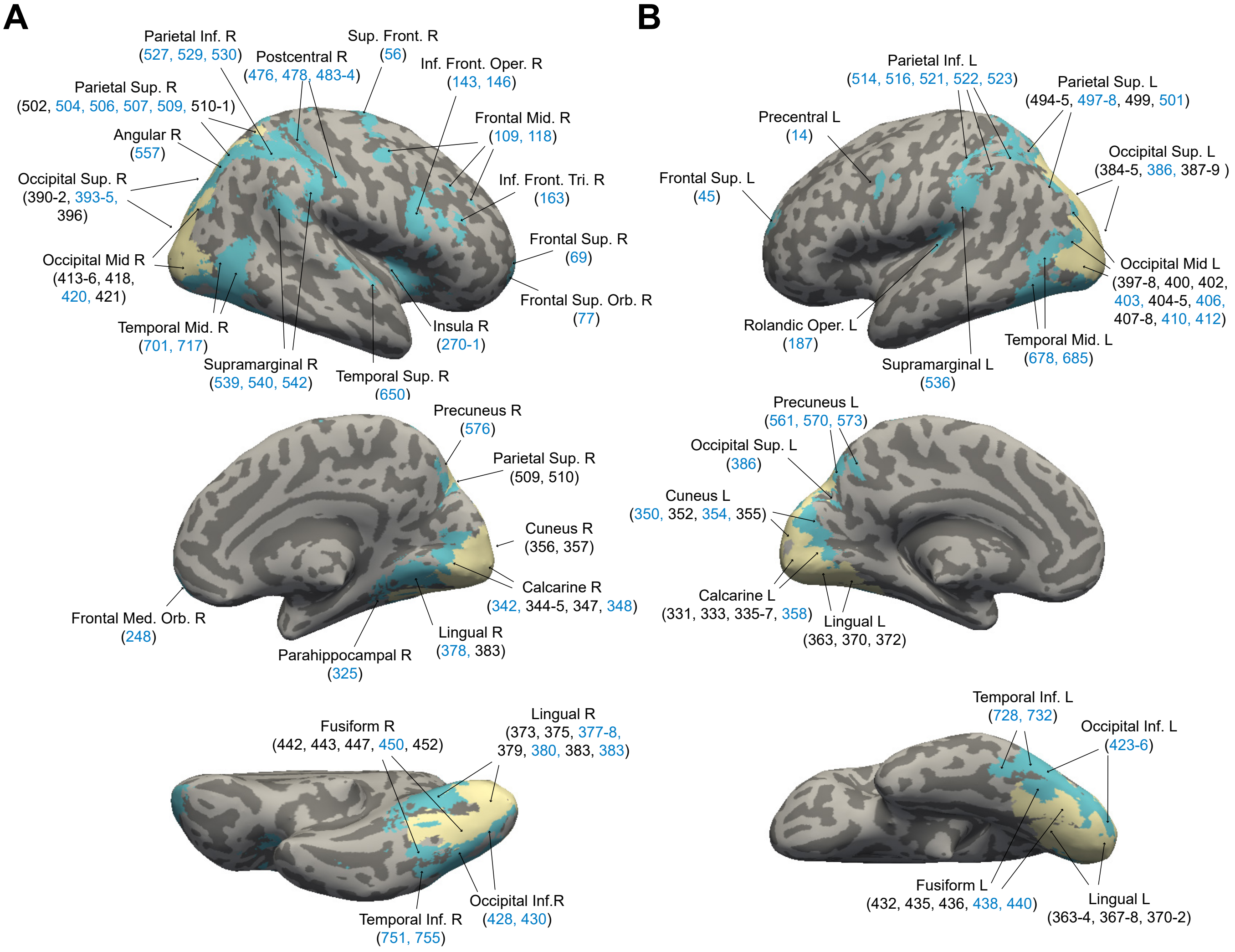

Figure S9: Overview of cortical parcels sensitive to community and/or selective for identity. Topographically organized regions in the visual cortex (in beige) are distinguished from other cortical regions (in blue). **A** Right hemisphere, lateral (top), medial (middle), and ventral views (bottom). **B** Left hemisphere, lateral (top), medial (middle), and ventral views (bottom).
